# Identification and analysis of unitary pseudogenes: historic and contemporary gene losses in humans and other primates

**DOI:** 10.1186/gb-2010-11-3-r26

**Published:** 2010-03-08

**Authors:** Zhengdong D Zhang, Adam Frankish, Toby Hunt, Jennifer Harrow, Mark Gerstein

**Affiliations:** 1 Department of Molecular Biophysics and Biochemistry, Yale University, New Haven, CT 06520, USA; 2 Wellcome Trust Sanger Institute, Hinxton, Cambridgeshire CB10 1HH, UK; 3 Interdepartmental Program in Computational Biology and Bioinformatics, Yale University, New Haven, CT 06520, USA; 4 Department of Computer Science, Yale University, New Haven, CT 06520, USA

## Abstract

**Background:**

Unitary pseudogenes are a class of unprocessed pseudogenes without functioning counterparts in the genome. They constitute only a small fraction of annotated pseudogenes in the human genome. However, as they represent distinct functional losses over time, they shed light on the unique features of humans in primate evolution.

**Results:**

We have developed a pipeline to detect human unitary pseudogenes through analyzing the global inventory of orthologs between the human genome and its mammalian relatives. We focus on gene losses along the human lineage after the divergence from rodents about 75 million years ago. In total, we identify 76 unitary pseudogenes, including previously annotated ones, and many novel ones. By comparing each of these to its functioning ortholog in other mammals, we can approximately date the creation of each unitary pseudogene (that is, the gene 'death date') and show that for our group of 76, the functional genes appear to be disabled at a fairly uniform rate throughout primate evolution - not all at once, correlated, for instance, with the 'Alu burst'. Furthermore, we identify 11 unitary pseudogenes that are polymorphic - that is, they have both nonfunctional and functional alleles currently segregating in the human population. Comparing them with their orthologs in other primates, we find that two of them are in fact pseudogenes in non-human primates, suggesting that they represent cases of a gene being resurrected in the human lineage.

**Conclusions:**

This analysis of unitary pseudogenes provides insights into the evolutionary constraints faced by different organisms and the timescales of functional gene loss in humans.

## Abstract 2

Novel human pseudogenes are identified that had previous functionality and their age is estimated. The rate of loss-of-function occurred uniformly.

## Background

Pseudogenes (*ψ*) are nongenic DNA segments that exhibit a high degree of sequence similarity to functional genes but contain disruptive defects. The initial pseudogenization of a functional gene is most likely a single mutagenic event that results in premature stop codons, abolished splice junctions, shifts to the coding frame, or impaired transcriptional regulatory sequences. Most pseudogenes are disabled copies of a functional 'parent' gene and can be classified as either processed or duplicated pseudogenes depending on whether they are generated by the retro-transposition of processed mRNA transcripts or the duplication of gene-containing DNA segments in the genome. Recently, the pseudogene complement of the human genome has been investigated both in gene family-specific studies [1-4] and in comprehensive surveys [5-7]. Of the approximately 20,000 pseudogenes identified in early studies, most, if not all, do not represent the extinction of a function as their 'parent' genes are intact and functional.

A third group of pseudogenes particularly relevant to functional analyses are unitary pseudogenes, which are unprocessed pseudogenes with no functional counterparts. They are generated by disruptive mutations occurring in functional genes and prevent them from being successfully transcribed or translated. They differ from duplicated pseudogenes in that the disabled gene had an established function rather than being a more recent copy of a functional gene. The initial analysis of the euchromatic sequence of the human genome identified 37 unitary pseudogene candidates [8]. In addition to unitary pseudogenes with fixed disruptive nucleotide substitutions, human genes with polymorphic disruptive sites that are currently segregating in the human population have also been indentified [8-10], and many of them provide the genetic bases of certain inheritable diseases [11]. Such gene deactivation, which happens *in situ *giving rise to a unitary pseudogene, results in a loss to the functional part of the genetic repertoire of the organism. Polymorphic pseudogenes are unlikely to become fixed in a population if the gene loss is deleterious. However, various evolutionary processes, such as genetic drift, migration (population bottleneck), and in some cases, natural selection, can lead to fixation. A number of genes are known to have been lost in the human lineage in comparison with other mammals [4,12-15].

In this study, we develop a novel comparative genomic approach to identify genes disabled *in situ *without a functional copy (unitary pseudogenes) using the absence of human proteins orthologous to their mouse counterparts as the signals of losses of well-established genes. Our method is able to systematically detect the sequence signature left by such genic losses, distinguishing true loss from mere loss of redundant genes following duplication or retrotransposition. We identify historic and contemporary losses of protein-coding genes in the human lineage since the last common ancestor of euarchontoglires (primates and rodents). In addition to pseudogenes in tandem gene families, we identify 76 losses of well-established genes in the human lineage since the common ancestor with mouse. Moreover, we also find 11 genes with polymorphic disruptive sites. This latter set represents gene losses on a very different timescale: the genic and pseudogenic alleles are segregating in the current human population and are subject to various evolutionary forces.

## Results

### Gene loss is indicated by the absence of orthologs

After a speciation event, the increasing divergence between two resultant species reflects the diminution in their genic orthology as gains and losses of genes gradually accumulate in each of them. Thus, the presence of genes unique to one species relative to another indicates either gene gains in one or gene losses in the other. In common with many other genomic features, genes in all species are in a state of flux during evolution. However, since all species are related to one another through speciation, gains and losses of genes in one species can be identified only relative to another. Based on this observation, we developed a pipeline that uses the orthologous relationship between genes from a pair of species to detect gene losses in one of them.

**Figure 1:**
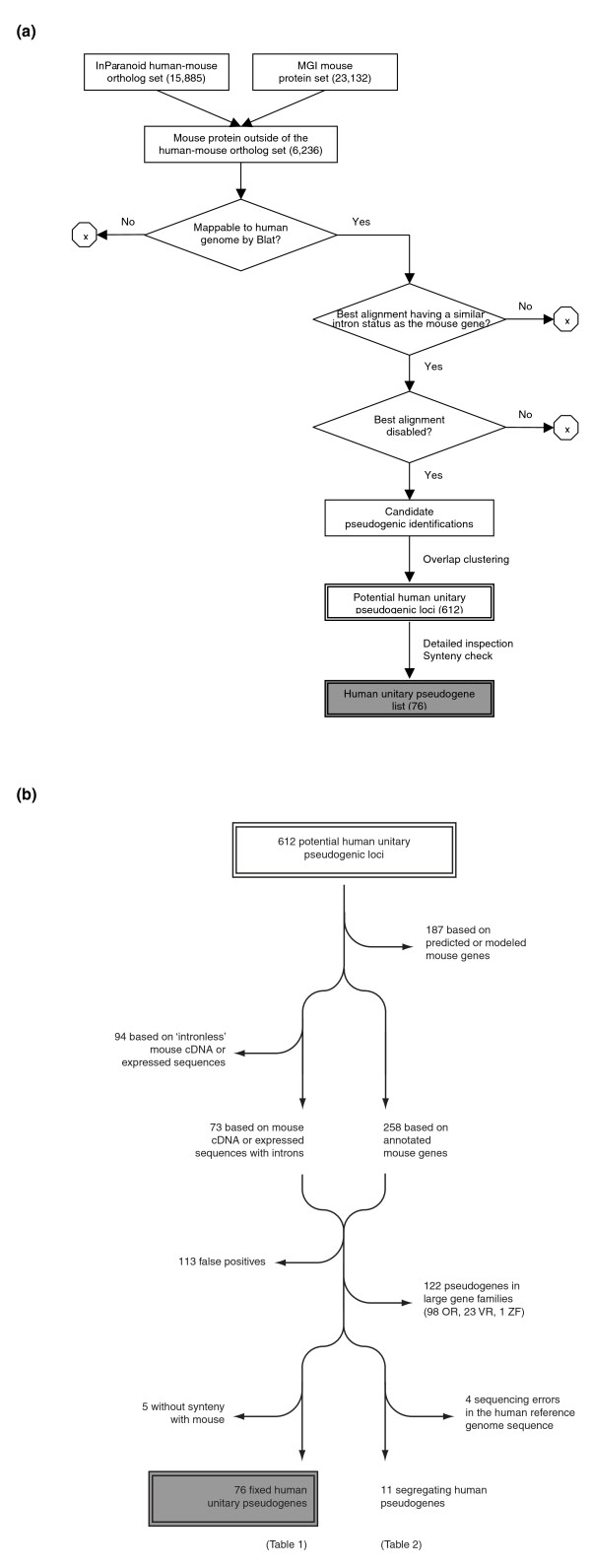
**Method for identifying human unitary pseudogenes in comparison to the mouse genome**. **(a) **The overall methodological flowchart. The number of entries in the input/output data set used at certain steps is shown in parentheses. **(b) **Detailed inspection and synteny check of the potential human unitary pseudogenic loci. Entries in the initial set of pseudogenic loci are removed based on various criteria at different steps. The final result - the unitary pseudogenes and the polymorphic pseudogenes in human - are listed in Tables 1 and 2. See the main text for details. MGI, Mouse Genome Informatics. OR, olfactory receptor; VR, vomeronasal receptor; ZF, zinc finger protein.

To take advantage of rich genomic annotation available for mouse, our study uses the mouse gene set as the reference to identify genes that have been lost in the human lineage since the divergence of these two species. Using the InParanoid [16] human-mouse orthologous gene set, we find 6,236 mouse proteins without discernible human orthologs. The presence of these unique mouse proteins indicates, most likely, both gene gains in the mouse lineage and gene losses in the human one. There are 2,005 unique mouse proteins that cannot be aligned to the human genome and thus are likely to be gene gains in the mouse. For the remaining unique mouse proteins that can be aligned, we found disruptions to the putative human coding sequences in 974 sequence alignments. Subsequent removal of redundancy reveals 612 potentially pseudogenic loci; 187 loci are removed from the list because they are identified based on predicted or modeled mouse genes, whose validity cannot be easily verified; 94 loci are also removed without further consideration as their identifications are based on unspliced mouse transcribed sequences labeled as 'expressed' or 'RIKEN cDNA' sequences. The filtering steps leave 258 loci based on annotated mouse genes and 73 of these are based on spliced mouse 'expressed' or 'RIKEN cDNA' sequences. Manual inspection of each of the remaining 331 pseudogenic loci removes 113 false positives (such as ones found in short, low-quality sequence alignments) and confirms the presence of 228 disabled human genes, which include 122 pseudogenes in large gene families, 81 possible fixed human unitary pseudogenes, and 15 likely segregating human pseudogenes. After removing five human fixed pseudogenes that are not in regions syntenic to those of their mouse orthologs and four segregating pseudogenes whose identifications are attributed to the sequence errors in the human reference genome, we identify 87 unitary pseudogenes, of which 76 are fixed and 11 still segregating in the human population (Figure 1b).

### Many genes were lost in the human lineage since the human-mouse divergence

Using the human-mouse genic orthology, we identify 228 pseudogenic loci - about 1% of the human gene catalog - in the human genome, which include 98 olfactory receptors, 23 vomeronasal receptors, and 1 zinc finger protein. The large number of olfactory receptors and vomeronasal receptors found in our study is consistent with previous observations [17,18]. These gene families form tandem gene clusters and have experienced copy number changes and complex local rearrangements. Because the dynamics of gene clusters make it difficult to unambiguously discern ortholog/paralog relationships between species, it is difficult to discern the 'unitary' status of the olfactory receptor/vomeronasal receptor/zinc finger pseudogenes (Table S1 in Additional file 1) and thus they are excluded from further analyses in this study.

We found 76 gene losses in the human lineage since the human-mouse divergence (Table 1; see Table S2 in Additional file 1 for more information). Of these, 31 are identified through uncharacterized mouse genes. Some are previously identified human unitary pseudogenes, such as pseudogenes of gulonolactone (L-) oxidase (*GULO*), an enzyme that produces the precursor to vitamin C [19], urate oxidase (*UOX*), an enzyme that catalyzes the oxidation of uric acid to allantoin [15], and Farnesoid × receptor beta, a nuclear receptor for lanosterol [4]. In addition, we also confirm the human-specific loss of cardiotrophin-2 (*CTF2*) due to a frameshift to its coding sequence caused by an 8-bp deletion [20], and hyaluronoglucosaminidase 6 (*HYAL6*) with two frameshift-causing deletions [21].

**Table 1:** Human unitary pseudogenes

Human unitary pseudogene genomic location	Mouse ortholog symbol	Mouse gene name
chr12+:110821507-110823878	*Adam1b*	a disintegrin and metallopeptidase domain 1b
chr8+:17371392-17373372	*Adam26B*	a disintegrin and metallopeptidase domain 26B
chr8-:39450156-39489335	*Adam3*	a disintegrin and metallopeptidase domain 3 (cyritestin)
chr8+:39299218-39358412	*Adam5*	a disintegrin and metallopeptidase domain 5
chr9-:103136199-103141451	*Acnat2*	acyl-coenzyme A amino acid N-acyltransferase 2
chr18+:54814947-54887164	*Acyl3*	acyltransferase 3 [RIKEN cDNA 5330437I02 gene]
chr1+:92304452-92305907	*Aytl1b*	acyltransferase like 1B
chr11+:71909632-71910345	*Art2b*	ADP-ribosyltransferase 2b
chr2+:201166115-201364602	*Aox3l1*	aldehyde oxidase 3-like 1
chr16+:2351147-2415839	*Abca17*	ATP-binding cassette, sub-family A (ABC1), member 17
chr1-:51789487-51812353	*Calr4*	calreticulin 4
chr16-:30823174-30826438	*Ctf2*	cardiotrophin 2
chr4-:123871155-123872802	*Cetn4*	centrin 4
chr19-:46006279-46009136	*Cyp2t4*	cytochrome P450, family 2, subfamily t, polypeptide 4
chr2-:178665477-178677441	*Cyct*	cytochrome c, testis
chr4-:68540001-68564082	*Desc4*	Desc4 [RIKEN cDNA 9930032O22 gene]
chr11-:67136888-67140266	*Doc2 g*	double C2, gamma
chr9+:35423704-35439561	*Feta*	Feta [RIKEN cDNA 4930417 M19 gene]
chr10-:114057930-114106344	*Gucy2 g*	guanylate cyclase 2 g
chr8:27473706-27502505	*Gulo*	gulonolactone (L-) oxidase
chr1-:226718541-226718916	*Hist3 h2ba*	histone cluster 3, H2ba
chr7+:123241442-123256569	*Hyal6*	hyaluronoglucosaminidase 6
chr9-:114761447-114764366	*Mup4*	major urinary protein 4
chr10+:81670064-81672769	*Mbl1*	mannose binding lectin (A) 1
chr6+:118061593-118072916	*Nepn*	nephrocan
chr3+:47028800-47029644	*Nradd*	neurotrophin receptor associated death domain
chr1+:115181467-115195621	*Nr1 h5*	nuclear receptor subfamily 1, group H, member 5
chrX+:101400687-101403403	*Prame*	preferentially expressed antigen in melanoma
chr1+:200404371-200425048	*Ptprv*	protein tyrosine phosphatase, receptor type, V
chr5+:140786050-140870922	*Pcdhgb8*	protocadherin gamma subfamily B, 8
chr19+:53875091-53876096	*Sec1*	secretory blood group 1
chr20-:1696610-1708642	*Sirpb3*	Sirpb3 [RIKEN cDNA F830045P16 gene]
chr2+:20449670-20459798	*Slc7a15*	solute carrier family 7 (cationic amino acid transporter, y+ system), member 15
chr4-:70692183-70714196	*Sult1d1*	sulfotransferase family 1D, member 1
chr7+:142844251-142845153	*Tas2r134*	taste receptor, type 2, member 134
chr17+:59285910-59292052	*Tcam1*	testicular cell adhesion molecule 1
chrX+:83901067-83903982	*Tex16*	testis expressed gene 16
chr14-:63882652-63893934	*Tex21*	testis expressed gene 21
chr8-:145268106-145414584	*Tssk5*	testis-specific serine kinase 5
chr17-:73756179-73757460	*Tha1*	threonine aldolase 1
chr1+:33704438-33707143	*Tlr12*	toll-like receptor 12
chr6:-132971083-132972109	*Taar3*	trace amine-associated receptor 3
chr6-:132957230-132958269	*Taar4*	trace amine-associated receptor 4
chr11+:3587708-3615320	*Trpc2*	transient receptor potential cation channel, subfamily C, member 2
chr4-:68314827-68322204	*Tmprss11c*	transmembrane protease, serine 11c
chr16-:2829662-2831734	*Tmprss8*	transmembrane protease, serine 8 (intestinal)
chr1-:84603696-84623086	*Uox*	urate oxidase

See Table S2 in Additional file 1 for the list of 29 human unitary pseudogenes identified using unannotated mouse gene transcripts.

**Figure 2:**
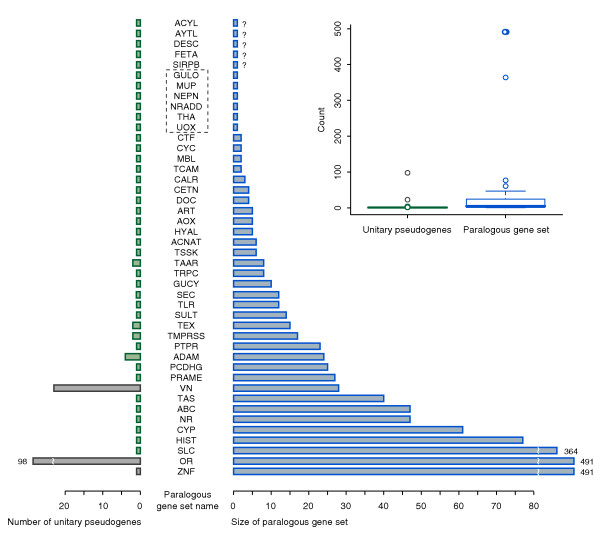
**The origin of human unitary pseudogenes in the paralogous gene sets**. The human unitary pseudogenes with annotation from orthologous mouse genes are assigned to human paralogous gene sets, whose names are shown in the middle. The number of human unitary pseudogenes in each paralogous gene set and the number of members in each paralogous gene set are plotted as green and blue bars, respectively. Five unitary pseudogenes with uninformative annotation are denoted with question marks. Unitary pseudogenes without close paralogs are enclosed by dashed lines. The unitary pseudogenes from the tandem gene families are indicated by gray bars. Inset: box plot of the number of human unitary pseudogenes in each paralogous gene set and the number of members in each paralogous gene set.

Most of the 76 gene losses occurred in gene families with multiple members: of the 47 examples that are orthologous to annotated mouse genes and whose synteny with their mouse orthologs can be identified with confidence; half of them are from gene families with more than six members (Figure 2). There is, however, no correlation between the size of gene families and the number of unitary pseudogenes from them. At one extreme, pseudogenes of GULO, major urinary protein (*MUP*), nephrocan (*NEPN*), neurotrophin receptor associated death domain (*NRADD*), threonine aldolase 1 (*THA1*), and UOX do not have any closely related paralogs. These genes are particularly intriguing as there are no alternatives with similar sequences and, as such, they represent unequivocal losses of biological functions. Below we examine NEPN and MUP in more detail as two case studies.

In a recent study, Mochida *et al*. showed NEPN is a secreted N-glycosylated protein inhibitor of transforming growth factor-β signaling in mouse and also identified putative *NEPN *gene orthologs in pig, dog, rat, and chicken [22]. The human ortholog was not found, and its absence was postulated to be a missed identification due to a lesser homology with its counterparts in other mammals. As this study and a previous one [23] demonstrate, however, despite the lack of a closely related homolog in the human genome, *NEPN *is a pseudogene not only in human but also in chimpanzee, gorilla, and rhesus with a shared coding sequence (CDS) disruptive mutation; thus, its inactivation occurred at least 30 millions of years ago, before the divergence between the catarrhines and the New World monkeys.

**Figure 3:**
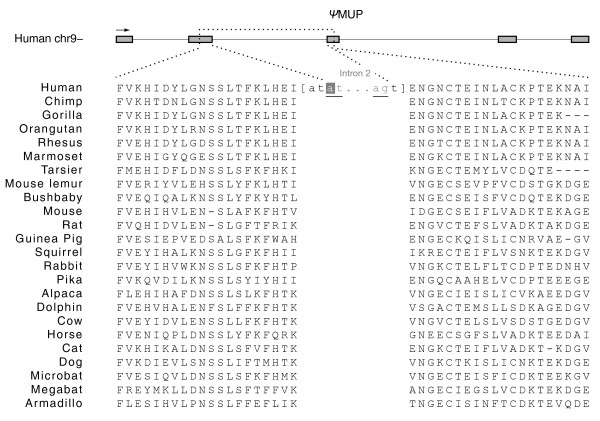
**The human-specific pseudogene of the major urinary protein**. A G-to-A nucleotide substitution (with the reverse highlight) at the donor site of the second intron (delineated by the underlined splicing sites) abolishes the ORF of the coding sequence. The sequence conservation is clearly discernable from the multiple sequence alignment of polypeptide sequences translated from partial exonic sequences upstream and downstream of the splicing junction of *MUP *from 24 species.

Except for *MUP *[24], which is a unitary pseudogene only in human, all other five genes - *GULO*, *NEPN*, *NRADD*, *THA1*, and *UOX *- were inactivated at least before the separation of human and chimpanzee (see below). Our study shows that human *MUP *was inactivated by a splice-junction mutation (GT to AT) located at the splice donor site of its second intron (Figure 3). This ORF-disrupting mutation in *MUP *is not seen in any other mammals whose genome sequences are available for examination. Using complete (or nearly complete) *MUP *gene sequences from human, chimpanzee, orangutan, rhesus and marmoset, we reconstruct the gene sequences at ancestral nodes in its primate phylogeny and calculate the *K*_A_/*K*_S _ratio along each lineage. The *K*_A_/*K*_S _ratio ranges from 0.36 to 0.58 and averages out to 0.54, an elevated value compared with 0.12, the median *K*_A_/*K*_S _ratio of protein-coding genes between human and mouse [25]. A recent study showed the MUP protein in mice is a pheromone ligand that promotes aggressive behaviors through its binding to the Vmn2r putative pheromone receptors (V2Rs) of the accessory olfactory neural pathway and, compared to other mammals being examined, there is a co-expansion of MUPs and V2Rs in mouse, rat, and opossum [24]. Our analysis shows all human V2Rs have been inactivated, corroborating previous studies, which revealed V2Rs are also lost in other primates [18,24]. Thus, the pseudogenization of human *MUP *and the overall accelerated nonsynonymous substitution rate in *MUP *of primates suggest it could be a direct result of the loss of the V2Rs, its specific receptors.

### Hydrolase-related activity and structure are enriched in human unitary pseudogenes

**Figure 4:**
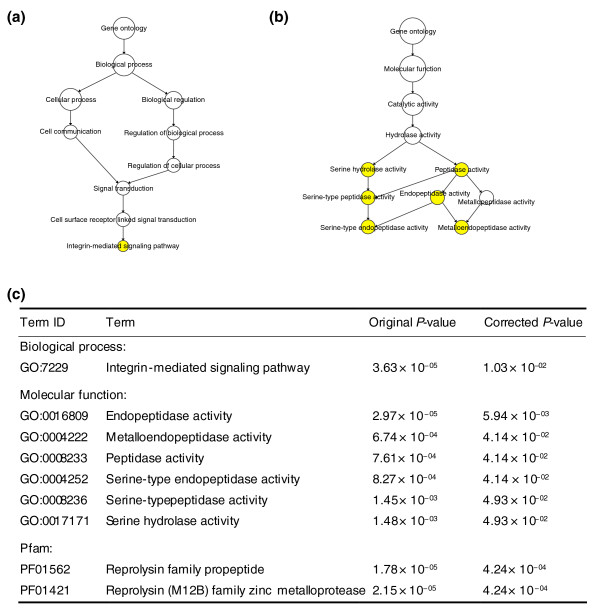
**Enrichment of Gene Ontology terms and Pfam domains in the human unitary pseudogene**. Enriched GO terms and their positions in the hierarchy of **(a) **biological process and **(b) **molecular function terms. Yellow nodes correspond to significant GO terms. **(c) ***P*-values for significant GO terms and Pfam domains.

Before pseudogenization, the protein products of these human unitary pseudogenes played diverse molecular functional roles in many different biological processes at various cellular locations as seen in their mouse counterparts. To determine whether there is an enrichment of labels in any of these three aspects of annotation, we test for Gene Ontology (GO) term association in the functional mouse counterparts of the human unitary pseudogenes on the GO hierarchy using Fisher's exact test. After correcting for multiple hypothesis tests to control the false discovery rate, we found significant enrichment of one biological process term, the integrin-mediated signaling pathway, and six molecular function terms, which are all specialized hydrolase activity (Figure 4a, b), among the mouse orthologs of the human unitary pseudogenes. The annotation shows that if functional, nine human unitary pseudogenes would encode for endopeptidases. Further examination shows five of them - transmembrane protease, serine 8 (intestinal) and 11, and three unnamed RIKEN cDNA genes - have the serine-type endopeptidase activity, and the other four - a disintegrin and metallopeptidase domain (ADAM) 1, 3, 5, and 26 - have the metalloendopeptidase activity. Protein domain analysis shows that two Pfam domains - reprolysin family propeptide and reprolysin (M12B) family zinc metalloprotease - are enriched in the human unitary pseudogenes (Figure 4c). Both of them are found in the ADAM unitary pseudogenes.

Compared with mouse, human has lost five testis-specific genes: testicular cell adhesion molecule 1 (*TCAM1*), testis expressed gene 16 (*TEX16*), testis expressed gene 21 (*TEX21*), testis-specific serine kinase 5 (*TSSK5*), and cytochrome c, testis (*CYCT*) [2]. The losses of these testis-specific genes in the human lineage may have affected the distinctive processes that occur in male germinal cells [26] and thus contributed to the differentiated fertility between two lineages.

### Gene loss has occurred throughout primate evolution

**Figure 5:**
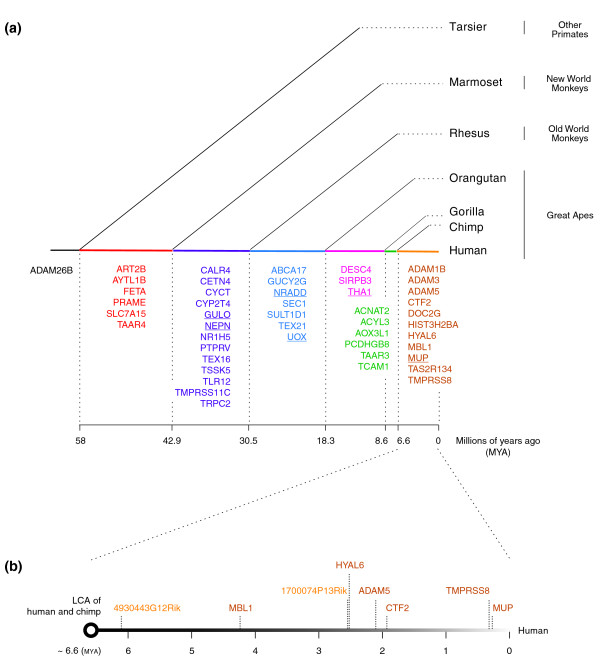
**Dating the pseudogenization events**. **(a) **Timing of the disruptive mutations that gave rise to human unitary pseudogenes by analyzing shared mutations. Only pseudogenes with annotations from orthologous mouse genes are shown. Ones without close paralogs are underlined. **(b) **Timing of several pseudogenization events that occurred in the human lineage after the human-chimp divergence. See Table S3 in Additional file 1 for the estimates and their standard errors. LCA, last common ancestor.

To estimate the time when functional genes were disabled to give rise to the human unitary pseudogenes, we identify the earliest shared ORF-disrupting mutations between humans and other mammals on the mammalian species tree. Very few pseudogenic mutations are shared outside of the primate clade. The most recent lineages where the occurrence of the pseudogenic mutations in the 47 annotated human unitary pseudogenes can generate their observed sharing pattern are illustrated on a primate phylogeny (Figure 5a). Such shared mutations indicate the pseudogenization events happened at every stage during primate evolution: from the human lineage alone to the last common ancestor of the great apes, the Old World monkeys, the New World monkeys, and the tarsiers.

One interesting case is the evolution of *NR1H5 *in primates. A previous study of the nuclear receptor pseudogenes [4] has shown that *NR1H5 *is a pseudogene in human, chimpanzee, and rhesus monkey with three (out of 14 in total) disruptive mutations - one frame-shift mutation and one splice-junction mutation in the very early part of the gene structure and one nonsense mutation at the end of the CDS - shared by these three primate species. In the same study, based on sequences from human, mouse, rat, and chicken, the silencing of *NR1H5 *was dated to be approximately 42 million years ago (MYA), which was slightly later than 42.9 MYA, the estimated time of divergence between the catarrhines and the New World monkeys [27]. However, because of the uncertainties in the estimates of both dates (for example, the 95% credibility interval of the divergence time estimation is from 36.1 to 51.1 MYA), it is not conclusive that the pseudogenization of *NR1H5 *occurred after the divergence between the catarrhines and the New World monkeys. To solve this problem, we identify *NR1H5 *in the recently published genomic sequences of marmoset, a New World monkey, and determine whether it contains any of the three pseudogenic mutations common to human, chimpanzee, and rhesus. Despite the fact that only the first one-third of the *NR1H5 *CDS can be found in marmoset due to the incompleteness of its genome assembly, the two important common disruptive mutations, whose positions are covered by the partial sequence identification, are absent. This finding suggests that the pseudogenization of *NR1H5 *in the human lineage occurred indeed after the divergence between the catarrhines and the New World monkeys.

Using current genome sequences of human, chimpanzee, gorilla, orangutan, rhesus, marmoset, and tarsier, we identify 11 genes - *ADAM3*, *CTF2*, *HIST3H2BA*, *MBL1*, *MUP*, *TMPRSS8*, *ADAM1B*, *ADAM5*, *DOC2G*, *HYAL6*, and *TAS2R134 *- with human-specific CDS disruptions, which occurred after the divergence of humans and chimpanzees. Based on our sequence analysis, however, we find the last five of them - *ADAM1B*, *ADAM5*, *DOC2G*, *HYAL6*, and *TAS2R134 *- are possibly also disabled in other primates with disruptions at different sites. Under the assumption that the neutral mutation rate has remained constant since the human-chimpanzee divergence at 6.6 MYA, we estimate the time in the hominid ancestor when the human-specific inactivation mutations appeared in the aforementioned 11 genes. The inactivation time of eight genes can be meaningfully calculated, and the estimates are plotted along the timeline from 6.6 MYA, when human and chimpanzee diverged, to the present (Figure 5b; Table S3 in Additional file 1). None of unitary pseudogenes seems to be generated by the insertion of an Alu sequence into the coding sequence of an ancestral functional gene. As the plot shows, unlike Alu sequences, which had an exceptional surge of activity around 40 MYA [28], the pseudogenization events occurred in a temporally random fashion - that is, there is no burst of gene losses during the human evolution since the human-chimpanzee divergence. This difference in their age distributions reflects the difference in underlying generative mechanisms.

### Some genes contain polymorphic disruptive sites and are segregating in the human population

Some of the pseudogenic loci are transcribed and, contrary to the genomic sequence, their mRNA transcript sequences lack the disruptive sites, suggesting they are functional genes. Such discrepancy potentially indicates the existence of polymorphic disruptive sites in those genes as the genomic DNA and the mRNA were obtained and sequenced from different individuals. After careful examination of both the genomic and the transcript sequences to ascertain their validity, we identified 11 human genes with polymorphic disruptive sites (Table 2). Such genes are extreme cases of genetic polymorphisms, as they have a nonfunctional pseudogenic allele segregating in the human population. Eight disruptive sites - four nonsense mutations and four 1-bp indels - have been catalogued in dbSNP. Three of them, all nonsense mutations, were included and typed in the HapMap Project [29], and the other five sites are near HapMap SNPs with a physical distance ranging from 20 bp to 1.7 kb (Table 2).

**Table 2:** Human polymorphic pseudogenes

Gene	CDS disruptive mutation		dbSNP ID^c^	HapMap SNP ID
			
	Change^a^	Location^b^		
Nonsense mutation				
*FBXL21*	taT (Y) → taA	chr5+:135,300,350	rs17169429 (+27)	rs17169429 (+27)
*FCGR2C*	Cag (Q) → Tag	chr1+:159,826,011	rs3933769 (-60)	rs3933769 (-60)
*GPR33*	Cga (R) → Tga	chr14-:31,022,505	rs17097921	rs17097921
*SEC22B*	Caa (Q) → Taa	chr1+:143,815,304	rs2794062	rs16826061 (+95)
*SERPINB11*	Gaa (E) → Taa	chr18+:59,530,818	rs4940595	rs4940595
*TAAR9*	Aaa (K) → Taa	chr6+:132,901,302	rs2842899	rs2842899
				
Frame-shift mutation				
*CASP12*	ΔCA	chr11-:104,268,394-5	rs497116 (-67)	rs497116 (-67)
*KRTAP7-1*	ΔT	chr21-:31123841	rs35359062	rs9982775 (-20)
*PSAPL1*	∇A	chr4-:7,487,457	rs58463471	rs4484302 (+441)
*TMEM158*	∇A	chr3-:45,242,396	rs11402022	rs33751 (+725)
*TPSB2*	ΔC	chr16-:1,219,240	rs2234647	rs2745145 (-1771)

^a^Base change, deletion, and insertion are denoted by '→', '∇', and 'Δ' respectively. ^b^The genomic location, based on the NCBI build 36 of the Human Reference Genome, includes the chromosome, the strand ('+' being forward and '-' reverse), and the coordinate of the base change. ^c^The identifier of the mutation as in the dbSNP (build 129). If a mutation is not included in the dbSNP, the identifier of the closest SNP and its distance (shown in parentheses) to the mutation are shown instead.

**Figure 6:**
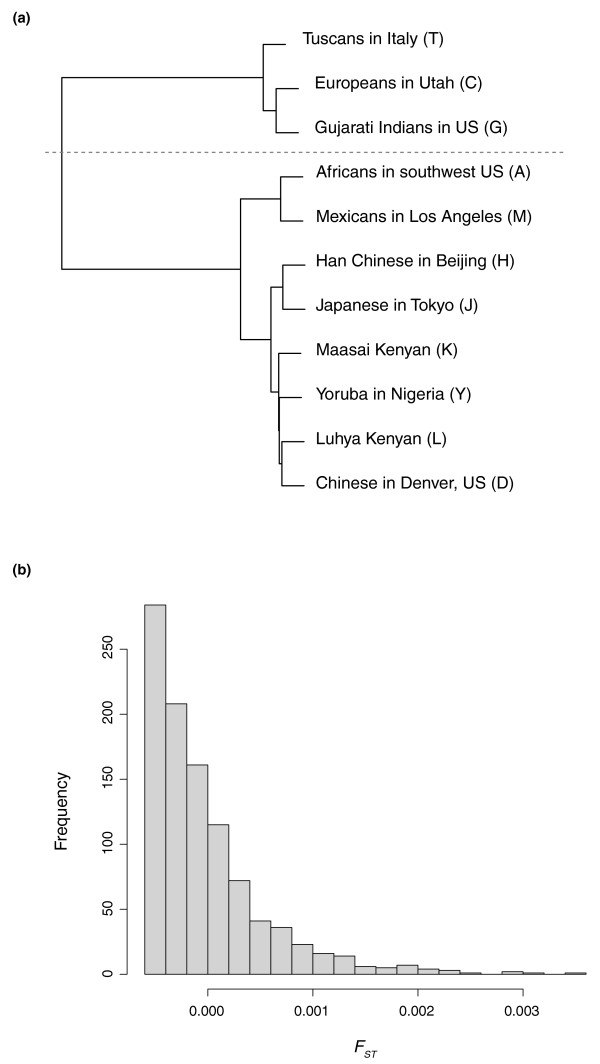
**Population structure analysis for SNP rs4940595**. **(a) **Hierarchical clustering of 11 populations using the *F*_ST _metric. Two subdivisions in the meta-population, as indicated by the dashed line, are clearly visible in the cluster. **(b) **Histogram of *F*_ST _from the permutation test using the population subdivisions as seen in (a).

Various genomic and genetic features of the HapMap SNPs rs17097921, rs4940595, and rs2842899 are summarized in Table 3 (see Table S4 in Additional file 1 for allele frequency information). Each of the nonsense alleles should effectively pseudogenize the gene, as all three SNPs are located in the early part of the coding sequences. Using the HapMap genotype data, several recent studies [30,31] scanned the human genome to detect positive selection in human populations. These three SNPs were not found to be under recent positive selection. Such negative results, however, could be caused by a lack of detection power due to a deficiency in data and/or method. The human reference alleles of all three SNPs are pseudogenic. The reference alleles in other primates are functional for rs17097921 but pseudogenic for both rs4940595 and rs2842899. Using the genotype and allele frequency data from the HapMap Project, we check for the Hardy-Weinberg equilibrium for the two alleles of each SNP in each population and all populations combined. Our statistical analysis shows that, in the meta-population, the two alleles, T/G, of rs4940595 are not at Hardy-Weinberg equilibrium (*χ*^2 ^goodness-of-fit test, degrees of freedom = 2, *χ*^2 ^= 8.659, *P *= 0.013). We calculate *F*_ST _between two populations to measure their difference (distance), and the *F*_ST _metric shows population subdivision in the meta-population. Hierarchical clustering groups 11 populations into two subdivisions: one composed of the Europeans in Utah, the Tuscans in Italy, and the Gujarati Indians in Houston, Texas, and the other the rest (Figure 6a). The *F*_ST _between these two subdivisions is 0.044, which is highly significant based on the permutation test (Figure 6b). Such population structure at rs4940595 - the difference in the allelic frequencies in different populations - could be the result, and thus a sign, of different selective regimes that the same allele at rs4940595 is subjected to in different population subdivisions.

**Table 3:** Polymorphic pseudogenes with the disruptive sites typed in the HapMap Project^a^

CDS disrupted gene	*GPR33*	*SERPINB11*	*TAAR9*
Disruptive mutation^b^	Cga (R) → Tga	Gaa (E) → Taa	Aaa (K) → Taa
dbSNP ID	rs17097921	rs4940595	rs2842899
Genomic location	chr14-:31,022,505	chr18+:59,530,818	chr6+:132,901,302
Disrupted codon position^c^	140 (332)	89 (388)	61 (344)
Reference allele in human	T	T	T
Reference allele in other primates^d^	C	T	T
Test statistic for HWE in the meta-population^e^	0.285 (*P *= 0.867)	8.659 (*P *= 0.013)	0.071 (*P *= 0.965)

^a^See Table S4 in Additional file 1 for allele frequency information. ^b^Both codons before and after the mutation (→) are shown with the affected base capitalized. The amino acid residue encoded by the codon is given in parentheses. ^c^The disrupted codon position in the coding sequence (CDS). The number of codons in the CDS is given in parentheses. ^d^Widely regarded as the ancestral allele. Other primates currently include chimp, orangutan, and macaque. ^e^The *χ*^2 ^goodness-of-fit test is used to test for the Hardy-Weinberg equilibrium (HWE) in the meta-population using the pooled genotype and allele frequency data.

## Discussion

The pseudogene complement of the human genome has been comprehensively surveyed in several early studies [5-7]. Using sequence similarity between the proteome and the (translated) genome as the signature, these studies found pseudogenic copies of functional genes that were generated after duplication or retrotransposition in the human genome. Such duplicated or processed pseudogenes are probably of little evolutionary significance, as the former are disabled soon after duplication and the latter 'dead on arrival' [32]. In this study, however, we systematically identify human unitary pseudogenes, a class of pseudogenes that are especially interesting as it is the functional genes themselves, not their genomic copies generated by duplication or retrotransposition, that have been disabled. Some human unitary pseudogenes have been identified on an individual basis when a particular gene or gene family was studied (see the references in Table S2 in Additional file 1). Using a comparative genomic approach, Zhu *et al*. [23] identified 26 losses of well-established genes in the human genome that were all lost at least 50 MYA after their birth. We compared our and their sets and found that in spite of using different methodological approaches, both studies had in common many gene losses in the human genome (Table S5 in Additional file 1).

**Figure 7:**
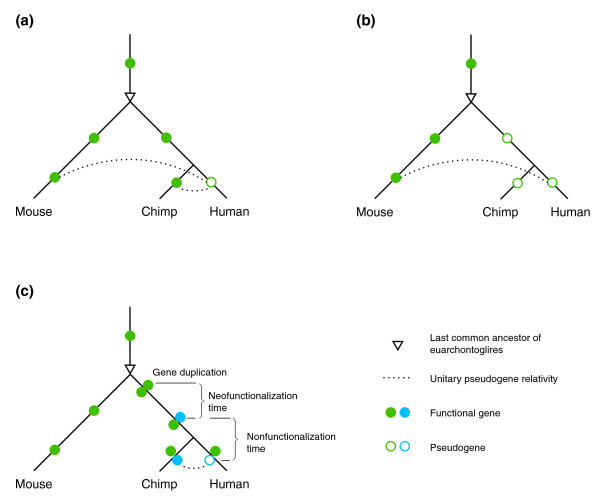
**Unitary pseudogene relativity**. Given the phylogeny of human, chimpanzee, and mouse, a human unitary pseudogenes can arise from a gene loss that occurred in different lineages, including: **(a) **the human lineage after the human-chimp divergence; **(b) **the human-ancestral lineage after the human-mouse divergence but before the human-chimp divergence; and under different circumstances, such as **(c) **loss of a subfunctionalized gene in the human lineage after a duplication event before the human-chimp divergence. Because the absence of a functional gene in a species is only identifiable through the comparison with another species that has the functional ortholog, the human unitary pseudogene can be identified in (a) by comparing the human gene set to either the chimp or the mouse set as both of them have the human ortholog. In (b, c), however, the human unitary pseudogene can only be identified by comparing the human gene set to one of either the mouse or chimp gene set, as the other one does not have the human ortholog given the evolutionary history of the gene under consideration.

To identify unitary pseudogenes in one species, we need a reference gene set from another species. This is not a mere operational convenience or necessity: unitary pseudogenes are conceptually comparative entities as speciation and gene duplication (and the possible subsequent gene death) are two separate events that most likely happen at different times. As a result, different sets of unitary pseudogenes in a species could be identified if reference gene sets from several species are used. For example, to identify human unitary pseudogenes, we can use mouse or chimpanzee gene sets. When the human gene loss happened after the human-chimp divergence and if the mouse and the chimp orthologs are both conserved, we have the same identifiable unitary pseudogene in human corresponding to its mouse or chimp ortholog (Figure 7a). If, however, the gene loss happened between the human-mouse and the human-chimp divergences and the mouse ortholog is conserved, the human unitary pseudogene is only meaningful and identifiable when the mouse gene set is used for the comparison (Figure 7b). In a slightly more complicated evolutionary scenario, if a gene was duplicated after the human-mouse divergence and its copy was successfully neo-functionalized (with substantial sequence change) before the human-chimp divergence and pseudogenized afterwards in the human lineage, the human unitary pseudogene is relative to, and identifiable by, its chimp ortholog (Figure 7c). Under this scenario, such human unitary pseudogenes - including human *ψMYH16 *- cannot be identified using the mouse protein/gene set and thus will be false negatives of the identification result (Table S6 in Additional file 1). The comparison between the human and chimpanzee genomic sequences has revealed a number of gene disruptions in humans [33].

Within a population, the pseudogenization of a gene does not happen instantaneously. Rather, after a disruptive mutation occurs, the alleles at the locus undergo a fixation process. Depending on the outcome, such a mutation is either fixed or lost. Thus, every gene loss goes through two stages: a polymorphic stage in the contemporary population subject to evolutionary forces; and a fixed stage freed from selective pressure. The fixed mutation becomes the base substitution in the species under study relative to the other and is identified through comparison of the genomes of two species. By comparing the human and the mouse genomes, we identify 76 fixed unitary pseudogenes. In addition, we identify 11 human genes with pseudogenic alleles, whose disruptive mutations include nonsense mutations and frameshifts. Our identification of polymorphic pseudogenes is by no means comprehensive as we search in the reference genome sequence for only the loci that are associated with both CDS disruptions and functional mRNA sequences. To obtain a comprehensive set of polymorphic pseudogenes, one approach would be to map variation sites in dbSNP to the reference genome and identify variations that can disrupt the ORF of known genes.

Being at a relatively early stage of pseudogenization, polymorphic pseudogenes in a population are subject to various evolutionary forces depending on the function of the normal alleles and the interaction between different genotypes and the environment. Since the loss of a single-copy gene is often deleterious and unlikely to be fixed in a population [34], it remains unclear under what circumstances genes were silenced and how the losses were tolerated and fixed in the ancestral population. It has been proposed that, under certain conditions, a gene could become disposable to the fitness of the organism if the function that it provides becomes redundant. When this happens, the pseudogenic allele could be fixed in the population by random genetic drift because the loss of the gene product did not constitute a disadvantage and, thus, there is little selection against the gene loss. This release from selective pressure is believed to be how the nonfunctionalization of L-gulono-γ-lactone oxidase gene could be fixed in humans and guinea pigs [13]: it has been hypothesized that the guinea pig and human ancestors subsisted on a naturally ascorbic acid-rich diet; therefore, the loss of the enzyme did not constitute a disadvantage.

On the other hand, as argued by the 'less is more' hypothesis, gene loss may serve as an engine of evolutionary change [35]. Instead of being a neutral event, the silencing of a gene could be advantageous to the organism and consequently sweep through the population to fixation - the kind of adaptive evolution illustrated by the inactivation of the α-1,3-galactosyltransferase gene in catarrhines [36], the CMP-N-acetylneuraminic acid hydroxylase gene [12], the olfactory receptor genes [17], and the sarcomeric myosin gene [14] in humans as there seems to be a correlation between pseudogenization and physiological/anatomic changes. In addition to these fixed unitary pseudogenes, studies have also shown that some null alleles confer a selective advantage for the polymorphic pseudogenes in the human population. For example, the chemokine receptor *CCR5 *gene in human has a pseudogenic allele with a 32-bp deletion. Homozygotes of this null allele are strongly protected from infection by various pathogens, including HIV, and heterozygotes receive some protection [9]. Another example is the caspase-12 gene. It has been shown that carriers of the caspase-12 pseudogene are more resistant to severe sepsis [37], and the null allele has spread through most of the human population within the past 100,000 years because of positive selection [38].

There are 6,236 Mouse Genome Informatics (MGI) mouse proteins and 6,020 Ensembl human proteins outside of the InParanoid-assigned human-mouse orthologs. Such an absence of orthology is a result of both gene deaths that generated unitary pseudogenes and gene births that gave rise to novel genes in both species. Using the absence of orthologs of mouse proteins in human as the signal, we identify 76 such losses of well-established genes in the human genome. Of the 2,005 human proteins that have no mouse orthologs and cannot be mapped to the mouse reference genome, 638 passed the quality control and thus are included in the current Ensembl release of the human protein set. Because they cannot be mapped to the genome of dog, the closest out-group of the human-mouse lineage with the best genomic sequences, we believe the reason for their lack of mouse orthologs is that they are novel human genes, not that their mouse orthologs have been deleted. If we take the 15,885 human-mouse orthologs assigned by InParanoid as the set of genes before the divergence between human and mouse, the unitary pseudogenes and the novel genes generated in the human lineage since the last common ancestor of euarchontoglires, approximately 75 MYA, represent, respectively, a loss and a gain of approximately 0.5% and 4% of the number of ancestral genes. Despite aforementioned examples of gene losses under positive selection, this striking skew toward gene birth indicates strongly that gene births are a more significant force for evolutionary change than gene losses. It also confirms the notion that as they represent functional losses to a species, unitary pseudogenes are expected to be rare.

**Figure 8:**
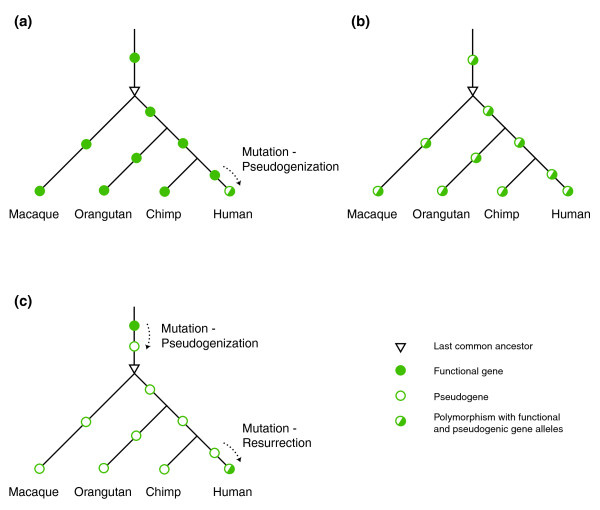
**Polymorphic pseudogenes in human populations**. **(a) **Human-specific pseudogenic polymorphism generated by gene inactivation. **(b) **Pseudogenic polymorphism since the last common ancestor. **(c) **Human-specific pseudogenic polymorphism generated by pseudogene resurrection.

The reference allele in other primates - which is widely taken as the ancestral state - of a human SNP can shed light on its emergence and evolution. The human reference alleles of three disruptive HapMap SNPs (Table 3) are pseudogenic, which cannot be otherwise given the method that we use to identify the polymorphic pseudogenes. As expected (Figure 8a), the reference allele of one SNP in non-human primates is functional. It is surprising, however, to find that the reference alleles of two of the SNPs in non-human primates are pseudogenic. One explanation is that these two loci in the common ancestor of human, chimp, orangutan, and macaque were also polymorphic and have been so in the descendent populations ever since (Figure 8b). If their pseudogenic alleles have risen to high frequencies in chimp, orangutan, and macaque, it is possible for these two loci to be typed as pseudogenic homozygotes (that is, the reference alleles) in all these three non-human primate populations. Polymorphisms at some HLA (human leukocyte antigen) loci are known examples of polymorphisms that have crossed speciation events, as these HLA loci are polymorphic in both human and chimp. This explanation, however, requires the polymorphisms at rs4940595 and rs2842899 to be very ancient, at least 30 million years old. Another explanation is that the pseudogenic alleles are indeed fixed in chimp, orangutan, macaque, and the last common ancestor between them and human, but the genes have been resurrected from the pseudogenic state in the human lineage (Figure 8c). This seemingly implausible resurrection event is believed to have happened to the human *IRGM *gene through a series of complex structural events after it became pseudogenized in the anthropoid common ancestor [39].

## Conclusions

Unitary pseudogenes are unprocessed pseudogenes with no functional counterparts. With complete genome sequences of model organisms, we have developed a novel method to detect such pseudogenes in a genome through analyzing the global inventory of orthologs between two organisms. Using this approach with very conservative cutoffs to look for gene losses along the human lineage after its divergence from rodents approximately 75 MYA, we identify 76 unitary pseudogenes in the human genome. As relics of genes, they shed particular light on the unique features of the human genome during evolution. By comparing orthologous sequences, we assign ages to primate unitary pseudogenes, and find that the former functional genes appear to have been disabled at a fairly uniform rate throughout primate evolution and not in a sudden burst. Furthermore, we find 11 polymorphic pseudogenes that have nonfunctional pseudogenic alleles currently segregating in the human population Comparing them with their orthologs in other primates, we find that two are in fact pseudogenes in non-human primates, suggesting that these actually represent cases of a gene that is in the process of being resurrected in the human lineage. Identification and analysis of human unitary pseudogenes afford unique insights into the evolution and dynamics of the human genic repertoire and the human genome at large.

## Materials and methods

### Identification of human unitary pseudogenes

The overall strategy of our approach is depicted in Figure 1a. To discover human unitary pseudogenes, we use mouse proteins as the reference. Because by definition a unitary pseudogene and a functional ortholog in a genome are mutually exclusive for a specific gene in another genome, we first identify mouse proteins that do not have human orthologs. To find such mouse proteins, we use the InParanoid human-mouse ortholog set (version 6.1, based on human Ensembl 43 and mouse MGI 12 December 2006 protein sets). InParanoid is used because it balances the false negative and false positive rates and was top-ranked as an orthology tool [40,41]. These mouse proteins are then mapped to the human reference genome (Hsap NCBI build 36.1, hg18) using BLAT [42] with its default parameters. If the best mapping of a mouse protein to the human genome gives a gene structure similar to that of the mouse gene, the mapped human genomic region is extracted and examined for disruptions (nonsense mutations and frameshifts) to the coding sequence using GeneWise [43].

Some of the initially discovered human pseudogenes are redundant as they could be identified by more than one mouse gene due to duplicated gene annotations or high sequence similarities among members of certain protein families. The redundancy is removed by clustering the initial set of pseudogenic candidates into pseudogenic loci based on the overlap among their genomic coordinates. These loci are grouped into four sets based on the annotation of the mouse proteins expressed from: named genes; cDNA/expressed sequences with introns; cDNA/expressed sequences without introns; and modeled/predicted genes. Given the low possibility for unitary pseudogenes to be intronless and the difficulty to assess the reliability of the modeled or predicted genes, the loci in the last two sets are excluded from further consideration.

Loci in the first two sets are carefully examined to ascertain their pseudogene status. Prior to manual annotation, all genomic sequences are sent to an automated analysis pipeline for similarity searches and *ab initio *gene predictions. The searches are run on a computer farm and stored in an Ensembl MySQL database using the Ensembl analysis pipeline system [44] and the results displayed in the Zmap genome viewer. Additional external predictions and annotation can be visualized in Zmap via a distributed annotation system (DAS). The otterlace annotation interface allows the user to build genes and edit annotations based on homology to aligned mRNA, expressed sequence tag and protein evidence by adding transcripts, exon coordinates, CDSs, gene names and descriptions, remarks and polyadenylation signals and sites [45].

All predicted unitary pseudogene loci are checked to ensure the validity of the orthologous mouse protein-coding gene, to verify the conservation of synteny between the human and mouse loci, and to confirm the pseudogenicity of the human locus. Mouse loci identified as orthologs to putative human unitary pseudogenes are fully manually annotated; that is, the complete gene structures and CDSs of all alternative splice variants are elucidated to confirm both the coding potential of the locus and the accuracy of the MGI annotated CDSs. Mouse loci identified as lacking a CDS are rejected as unitary pseudogenes. Conservation of synteny between mouse and human orthologs is established by the identification of conserved flanking loci in both the Zmap viewer and Ensembl MultiContig View. Where the position of the putative orthologs is not conserved, the human locus is rejected as a unitary pseudogene. Finally, the putative human unitary pseudogene locus is fully manually annotated. Loci are confirmed as unitary pseudogenes where the alignment of the orthologous mouse protein sequence indicates a CDS disruption (premature stop, frame-shift or truncation) fixed in the human genome.

We also identify several cases where the ORF of a gene is disrupted in the human reference genome sequence but locus-specific transcripts lack the disrupting mutation. Such a contradiction may be a result of polymorphism in the human population, as the genomic DNA and the mRNA were obtained from different individuals. However, in some cases an apparent error in the genomic sequence appears responsible. To identify and remove false positives, we check the validity of the base call under consideration in the human reference genome by examining the sequences of the reads in the trace archive. We confirm the transcript sequence by multiple independent copies available in GenBank. All errors in the genome sequence were reported to the Genome Reference Consortium.

### Identification of orthologous genic or pseudogenic sequences in 43 species

We examine 44 vertebrates for genic or pseudogenic sequences orthologous and syntenic to human unitary pseudogenes. The organism, release version and time of the genomic sequence download from the Ensembl database are listed in Table S7 in Additional file 1.

To identify orthologous and syntenic sequences, we first use the Fetch Alignments tool of Galaxy [46] to extract 'stitched' blocks of the alignment of the above 44 genomic sequences for each of the 76 human unitary pseudogenes in the human genome. Using the global multiple sequence alignment ensures the orthology and the synteny of mapped genomic sequences among species. The sequences in the alignment blocks are then mapped back using BLAT to their corresponding genomes to recover any sequences not included in the alignments. The subsequences corresponding to the 76 human unitary pseudogenes in the 44 genomes are extracted from the start minus 5 kb and the end plus 5 kb of the BLAT alignments. The mouse protein sequences are then aligned to the corresponding genomic subsequences using GeneWise to identify their orthologs in the 44 genomes.

### Functional and structural analyses of human unitary pseudogenes

For functional and structural analyses, we search for GO terms and Pfam domains that are over-represented within the human unitary pseudogenes. Because pseudogenes are nonfunctional and thus not included in the human gene annotation set, such analyses cannot be performed directly. To circumvent this problem, we use the 76 mouse functional orthologs of human unitary pseudogenes as their proxies. To perform the analyses, we combine all human genes and the 76 mouse genes into one gene list and retrieve their GO and Pfam annotations from Ensembl. BiNGO [47] is used to test the 76 mouse genes in comparison with the combined gene list for GO term association on the GO hierarchy. We also test for over-representation of Pfam domains using the standard hypergeometric test with subsequent false discovery rate correction for multiple hypotheses testing.

### Estimation of the nonfunctionalization time of a human-specific unitary pseudogene

To estimate the nonfunctionalization time (*T*_N_) of a unitary pseudogene, we use the method devised by Chou *et al*. [12]. It assumes that non-synonymous mutations are selected against until the gene is inactivated; thereafter, mutations at both synonymous and non-synonymous sites accumulate at the neutral mutation rate. Sequences orthologous to the human pseudogene from mouse and rat (and other organisms if available) are used in the calculation, as the quantification of lineage-specific mutation rates at synonymous and non-synonymous sites remote from the inactivating deletion provides the information necessary for the calculation. Given this assumption, the following equality holds:

in which *T *is the time since the last common ancestor of human and chimpanzee (approximately 6.6 MYA [27]), *T*_N _is the time since the unitary pseudogene inactivation to be estimated, *r*_S1 _= *K*_S1_/*T *is the synonymous substitution rate in the human lineage,  is the average *K*_A_/*K*_S _ratio in all non-human lineages, and *K*_A1 _is the nonsynonymous substitutions per nonsynonymous site in the human lineage. Rearrange the equation above, we have:

in which *ω*_1 _is the *K*_A_/*K*_S _ratio in the human lineage. When only a small number of species are used to estimate *T*_N_, its estimated value should be viewed with caution.

## Abbreviations

ADAM: a disintegrin and metallopeptidase domain; CDS: coding sequence; CTF: cardiotrophin; GO: Gene Ontology; GULO: gulonolactone (L-) oxidase; HYAL: hyaluronoglucosaminidase; MGI: Mouse Genome Informatics; MUP: major urinary protein; MYA: million of years ago; ORF: open reading frame; SNP: single nucleotide polymorphism; UOX: urate oxidase; V2R: Vmn2r putative pheromone receptor.

## Authors' contributions

Both ZDZ and MG conceived of the study. ZDZ designed and built the unitary pseudogene identification pipeline, carried out the downstream analyses of unitary pseudogenes in humans, and drafted the manuscript. AF, TH, and JH performed the manual examination of the predicted human unitary pseudogenic loci. MG participated in revision of the manuscript. All authors read and approved the final manuscript.

## Supplementary Material

Additional file 1 This file contains seven supplementary tables showing detailed results and datasets used in this study. Click here for file

## References

[B1] Glusman G Yanai I Rubin I Lancet D The complete human olfactory subgenome. Genome Res 2001 11 685 702 10.1101/gr.171001 11337468

[B2] Zhang Z Gerstein M The human genome has 49 cytochrome c pseudogenes, including a relic of a primordial gene that still functions in mouse. Gene 2003 312 61 72 10.1016/S0378-1119(03)00579-1 12909341

[B3] Zhang Z Harrison P Gerstein M Identification and analysis of over 2000 ribosomal protein pseudogenes in the human genome. Genome Res 2002 12 1466 1482 10.1101/gr.331902 12368239 PMC187539

[B4] Zhang ZD Cayting P Weinstock G Gerstein M Analysis of nuclear receptor pseudogenes in vertebrates: how the silent tell their stories. Mol Biol Evol 2008 25 131 143 10.1093/molbev/msm251 18065488

[B5] Ohshima K Hattori M Yada T Gojobori T Sakaki Y Okada N Whole-genome screening indicates a possible burst of formation of processed pseudogenes and Alu repeats by particular L1 subfamilies in ancestral primates. Genome Biol 2003 4 R74 10.1186/gb-2003-4-11-r74 14611660 PMC329124

[B6] Torrents D Suyama M Zdobnov E Bork P A genome-wide survey of human pseudogenes. Genome Res 2003 13 2559 2567 10.1101/gr.1455503 14656963 PMC403797

[B7] Zhang Z Harrison PM Liu Y Gerstein M Millions of years of evolution preserved: a comprehensive catalog of the processed pseudogenes in the human genome. Genome Res 2003 13 2541 2558 10.1101/gr.1429003 14656962 PMC403796

[B8] The International Human Genome Sequencing Consortium Finishing the euchromatic sequence of the human genome. Nature 2004 431 931 945 10.1038/nature03001 15496913

[B9] Dean M Carrington M Winkler C Huttley GA Smith MW Allikmets R Goedert JJ Buchbinder SP Vittinghoff E Gomperts E Donfield S Vlahov D Kaslow R Saah A Rinaldo C Detels R O'Brien SJ Genetic restriction of HIV-1 infection and progression to AIDS by a deletion allele of the CKR5 structural gene. Hemophilia Growth and Development Study, Multicenter AIDS Cohort Study, Multicenter Hemophilia Cohort Study, San Francisco City Cohort, ALIVE Study. Science 1996 273 1856 1862 10.1126/science.273.5283.1856 8791590

[B10] Tournamille C Colin Y Cartron JP Le Van Kim C Disruption of a GATA motif in the Duffy gene promoter abolishes erythroid gene expression in Duffy-negative individuals. Nat Genet 1995 10 224 228 10.1038/ng0695-224 7663520

[B11] Stenson PD Mort M Ball EV Howells K Phillips AD Thomas NS Cooper DN The Human Gene Mutation Database: 2008 update. Genome Med 2009 1 13 10.1186/gm13 19348700 PMC2651586

[B12] Chou HH Hayakawa T Diaz S Krings M Indriati E Leakey M Paabo S Satta Y Takahata N Varki A Inactivation of CMP-N-acetylneuraminic acid hydroxylase occurred prior to brain expansion during human evolution. Proc Natl Acad Sci USA 2002 99 11736 11741 10.1073/pnas.182257399 12192086 PMC129338

[B13] Koshizaka T Nishikimi M Ozawa T Yagi K Isolation and sequence analysis of a complementary DNA encoding rat liver L-gulono-gamma-lactone oxidase, a key enzyme for L-ascorbic acid biosynthesis. J Biol Chem 1988 263 1619 1621 3338984

[B14] Stedman HH Kozyak BW Nelson A Thesier DM Su LT Low DW Bridges CR Shrager JB Minugh-Purvis N Mitchell MA Myosin gene mutation correlates with anatomical changes in the human lineage. Nature 2004 428 415 418 10.1038/nature02358 15042088

[B15] Wu XW Lee CC Muzny DM Caskey CT Urate oxidase: primary structure and evolutionary implications. Proc Natl Acad Sci USA 1989 86 9412 9416 10.1073/pnas.86.23.9412 2594778 PMC298506

[B16] Berglund AC Sjolund E Ostlund G Sonnhammer EL InParanoid 6: eukaryotic ortholog clusters with inparalogs. Nucleic Acids Res 2008 36 D263 266 10.1093/nar/gkm1020 18055500 PMC2238924

[B17] Gilad Y Man O Paabo S Lancet D Human specific loss of olfactory receptor genes. Proc Natl Acad Sci USA 2003 100 3324 3327 10.1073/pnas.0535697100 12612342 PMC152291

[B18] Young JM Trask BJ V2R gene families degenerated in primates, dog and cow, but expanded in opossum. Trends Genet 2007 23 212 215 10.1016/j.tig.2007.03.004 17382427

[B19] Nishikimi M Fukuyama R Minoshima S Shimizu N Yagi K Cloning and chromosomal mapping of the human nonfunctional gene for L-gulono-gamma-lactone oxidase, the enzyme for L-ascorbic acid biosynthesis missing in man. J Biol Chem 1994 269 13685 13688 8175804

[B20] Derouet D Rousseau F Alfonsi F Froger J Hermann J Barbier F Perret D Diveu C Guillet C Preisser L Dumont A Barbado M Morel A deLapeyrière O Gascan H Chevalier S Neuropoietin, a new IL-6-related cytokine signaling through the ciliary neurotrophic factor receptor. Proc Natl Acad Sci USA 2004 101 4827 4832 10.1073/pnas.0306178101 15051883 PMC387333

[B21] Csoka AB Scherer SW Stern R Expression analysis of six paralogous human hyaluronidase genes clustered on chromosomes 3p21 and 7q31. Genomics 1999 60 356 361 10.1006/geno.1999.5876 10493834

[B22] Mochida Y Parisuthiman D Kaku M Hanai J Sukhatme VP Yamauchi M Nephrocan, a novel member of the small leucine-rich repeat protein family, is an inhibitor of transforming growth factor-beta signaling. J Biol Chem 2006 281 36044 36051 10.1074/jbc.M604787200 16990280

[B23] Zhu J Sanborn JZ Diekhans M Lowe CB Pringle TH Haussler D Comparative genomics search for losses of long-established genes on the human lineage. PLoS Comput Biol 2007 3 e247 10.1371/journal.pcbi.0030247 18085818 PMC2134963

[B24] Chamero P Marton TF Logan DW Flanagan K Cruz JR Saghatelian A Cravatt BF Stowers L Identification of protein pheromones that promote aggressive behaviour. Nature 2007 450 899 902 10.1038/nature05997 18064011

[B25] Mouse Genome Sequencing Consortium Waterston RH Lindblad-Toh K Birney E Rogers J Abril JF Agarwal P Agarwala R Ainscough R Alexandersson M An P Antonarakis SE Attwood J Baertsch R Bailey J Barlow K Beck S Berry E Birren B Bloom T Bork P Botcherby M Bray N Brent MR Brown DG Brown SD Bult C Burton J Butler J Campbell RD Initial sequencing and comparative analysis of the mouse genome. Nature 2002 420 520 562 10.1038/nature01262 12466850

[B26] Grimes SR Testis-specific transcriptional control. Gene 2004 343 11 22 10.1016/j.gene.2004.08.021 15563828

[B27] Steiper ME Young NM Primate molecular divergence dates. Mol Phylogenet Evol 2006 41 384 394 10.1016/j.ympev.2006.05.021 16815047

[B28] The International Human Genome Sequencing Consortium Initial sequencing and analysis of the human genome. Nature 2001 409 860 921 10.1038/35057062 11237011

[B29] The International HapMap Consortium A haplotype map of the human genome. Nature 2005 437 1299 1320 10.1038/nature04226 16255080 PMC1880871

[B30] Sabeti PC Varilly P Fry B Lohmueller J Hostetter E Cotsapas C Xie X Byrne EH McCarroll SA Gaudet R Schaffner SF Lander ES International HapMap Consortium Frazer KA Ballinger DG Cox DR Hinds DA Stuve LL Gibbs RA Belmont JW Boudreau A Hardenbol P Leal SM Pasternak S Wheeler DA Willis TD Yu F Yang H Zeng C Gao Y Genome-wide detection and characterization of positive selection in human populations. Nature 2007 449 913 918 10.1038/nature06250 17943131 PMC2687721

[B31] Voight BF Kudaravalli S Wen X Pritchard JK A map of recent positive selection in the human genome. PLoS Biol 2006 4 e72 10.1371/journal.pbio.0040072 16494531 PMC1382018

[B32] Lynch M Conery JS The evolutionary fate and consequences of duplicate genes. Science 2000 290 1151 1155 10.1126/science.290.5494.1151 11073452

[B33] The Chimpanzee Sequencing and Analysis Consortium Initial sequence of the chimpanzee genome and comparison with the human genome. Nature 2005 437 69 87 10.1038/nature04072 16136131

[B34] Graur D Li W-H Fundamentals of Molecular Evolution 2000 2 Sunderland, MA: Sinauer Associates, Inc

[B35] Olson MV When less is more: gene loss as an engine of evolutionary change. Am J Hum Genet 1999 64 18 23 10.1086/302219 9915938 PMC1377697

[B36] Galili U Swanson K Gene sequences suggest inactivation of alpha-1,3-galactosyltransferase in catarrhines after the divergence of apes from monkeys. Proc Natl Acad Sci USA 1991 88 7401 7404 10.1073/pnas.88.16.7401 1908095 PMC52303

[B37] Saleh M Vaillancourt JP Graham RK Huyck M Srinivasula SM Alnemri ES Steinberg MH Nolan V Baldwin CT Hotchkiss RS Buchman TG Zehnbauer BA Hayden MR Farrer LA Roy S Nicholson DW Differential modulation of endotoxin responsiveness by human caspase-12 polymorphisms. Nature 2004 429 75 79 10.1038/nature02451 15129283

[B38] Xue Y Daly A Yngvadottir B Liu M Coop G Kim Y Sabeti P Chen Y Stalker J Huckle E Burton J Leonard S Rogers J Tyler-Smith C Spread of an inactive form of caspase-12 in humans is due to recent positive selection. Am J Hum Genet 2006 78 659 670 10.1086/503116 16532395 PMC1424700

[B39] Bekpen C Marques-Bonet T Alkan C Antonacci F Leogrande MB Ventura M Kidd JM Siswara P Howard JC Eichler EE Death and resurrection of the human IRGM gene. PLoS Genet 2009 5 e1000403 10.1371/journal.pgen.1000403 19266026 PMC2644816

[B40] Chen F Mackey AJ Vermunt JK Roos DS Assessing performance of orthology detection strategies applied to eukaryotic genomes. PLoS One 2007 2 e383 10.1371/journal.pone.0000383 17440619 PMC1849888

[B41] Hulsen T Huynen MA de Vlieg J Groenen PM Benchmarking ortholog identification methods using functional genomics data. Genome Biol 2006 7 R31 10.1186/gb-2006-7-4-r31 16613613 PMC1557999

[B42] Kent WJ BLAT - the BLAST-like alignment tool. Genome Res 2002 12 656 664 11932250 10.1101/gr.229202 PMC187518

[B43] Birney E Clamp M Durbin R GeneWise and Genomewise. Genome Res 2004 14 988 995 10.1101/gr.1865504 15123596 PMC479130

[B44] Searle SM Gilbert J Iyer V Clamp M The otter annotation system. Genome Res 2004 14 963 970 10.1101/gr.1864804 15123593 PMC479127

[B45] Harrow J Denoeud F Frankish A Reymond A Chen CK Chrast J Lagarde J Gilbert JG Storey R Swarbreck D Rossier C Ucla C Hubbard T Antonarakis SE Guigo R GENCODE: producing a reference annotation for ENCODE. Genome Biol 2006 7 Suppl 1 S4.1 S4.9 10.1186/gb-2006-7-s1-s4 PMC1810553 16925838

[B46] Galaxy http://galaxy.psu.edu/

[B47] Maere S Heymans K Kuiper M BiNGO: a Cytoscape plugin to assess overrepresentation of gene ontology categories in biological networks. Bioinformatics 2005 21 3448 3449 10.1093/bioinformatics/bti551 15972284

[B48] Edwards DR Handsley MM Pennington CJ The ADAM metalloproteinases. Mol Aspects Med 2008 29 258 289 10.1016/j.mam.2008.08.001 18762209 PMC7112278

[B49] Hunt MC Alexson SE Novel functions of acyl-CoA thioesterases and acyltransferases as auxiliary enzymes in peroxisomal lipid metabolism. Prog Lipid Res 2008 47 405 421 10.1016/j.plipres.2008.05.001 18538142

[B50] Levy I Wu YQ Roeckel N Bulle F Pawlak A Siegrist S Mattei MG Guellaen G Human testis specifically expresses a homologue of the rodent T lymphocytes RT6 mRNA. FEBS Lett 1996 382 276 280 10.1016/0014-5793(96)00183-4 8605984

[B51] Garattini E Mendel R Romao MJ Wright R Terao M Mammalian molybdo-flavoenzymes, an expanding family of proteins: structure, genetics, regulation, function and pathophysiology. Biochem J 2003 372 15 32 10.1042/BJ20030121 12578558 PMC1223366

[B52] Piehler AP Wenzel JJ Olstad OK Haug KB Kierulf P Kaminski WE The human ortholog of the rodent testis-specific ABC transporter Abca17 is a ubiquitously expressed pseudogene (ABCA17P) and shares a common 5' end with ABCA3. BMC Mol Biol 2006 7 28 10.1186/1471-2199-7-28 16968533 PMC1579226

[B53] Csoka AB Frost GI Stern R The six hyaluronidase-like genes in the human and mouse genomes. Matrix Biol 2001 20 499 508 10.1016/S0945-053X(01)00172-X 11731267

[B54] Guo N Mogues T Weremowicz S Morton CC Sastry KN The human ortholog of rhesus mannose-binding protein-A gene is an expressed pseudogene that localizes to chromosome 10. Mamm Genome 1998 9 246 249 10.1007/s003359900735 9501312

[B55] Birtle Z Goodstadt L Ponting C Duplication and positive selection among hominin-specific PRAME genes. BMC Genomics 2005 6 120 10.1186/1471-2164-6-120 16159394 PMC1262708

[B56] Kelly RJ Rouquier S Giorgi D Lennon GG Lowe JB Sequence and expression of a candidate for the human Secretor blood group alpha(1,2)fucosyltransferase gene (FUT2). Homozygosity for an enzyme-inactivating nonsense mutation commonly correlates with the non-secretor phenotype. J Biol Chem 1995 270 4640 4649 10.1074/jbc.270.9.4640 7876235

[B57] Meinl W Glatt H Structure and localization of the human SULT1B1 gene: neighborhood to SULT1E1 and a SULT1D pseudogene. Biochem Biophys Res Commun 2001 288 855 862 10.1006/bbrc.2001.5829 11688987

[B58] Caenepeel S Charydczak G Sudarsanam S Hunter T Manning G The mouse kinome: discovery and comparative genomics of all mouse protein kinases. Proc Natl Acad Sci USA 2004 101 11707 11712 10.1073/pnas.0306880101 15289607 PMC511041

[B59] Edgar AJ Mice have a transcribed L-threonine aldolase/GLY1 gene, but the human GLY1 gene is a non-processed pseudogene. BMC Genomics 2005 6 32 10.1186/1471-2164-6-32 15757516 PMC555945

[B60] Roach JC Glusman G Rowen L Kaur A Purcell MK Smith KD Hood LE Aderem A The evolution of vertebrate Toll-like receptors. Proc Natl Acad Sci USA 2005 102 9577 9582 10.1073/pnas.0502272102 15976025 PMC1172252

[B61] Lindemann L Ebeling M Kratochwil NA Bunzow JR Grandy DK Hoener MC Trace amine-associated receptors form structurally and functionally distinct subfamilies of novel G protein-coupled receptors. Genomics 2005 85 372 385 10.1016/j.ygeno.2004.11.010 15718104

[B62] Wes PD Chevesich J Jeromin A Rosenberg C Stetten G Montell C TRPC1, a human homolog of a *Drosophila *store-operated channel. Proc Natl Acad Sci USA 1995 92 9652 9656 10.1073/pnas.92.21.9652 7568191 PMC40860

[B63] Piehler AP Hellum M Wenzel JJ Kaminski E Haug KB Kierulf P Kaminski WE The human ABC transporter pseudogene family: Evidence for transcription and gene-pseudogene interference. BMC Genomics 2008 9 165 10.1186/1471-2164-9-165 18405356 PMC2329642

[B64] Graw J Klopp N Loster J Soewarto D Fuchs H Becker-Follmann J Reis A Wolf E Balling R Habre de Angelis M Ethylnitrosourea-induced mutation in mice leads to the expression of a novel protein in the eye and to dominant cataracts. Genetics 2001 157 1313 1320 11238416 10.1093/genetics/157.3.1313 PMC1461562

[B65] Sheng J Ding X Identification of human genes related to olfactory-specific CYP2G1. Biochem Biophys Res Commun 1996 218 570 574 10.1006/bbrc.1996.0101 8561797

[B66] Steinmetz M Moore KW Frelinger JG Sher BT Shen FW Boyse EA Hood L A pseudogene homologous to mouse transplantation antigens: transplantation antigens are encoded by eight exons that correlate with protein domains. Cell 1981 25 683 692 10.1016/0092-8674(81)90175-6 6895187

