# Supplementary material for: Identification and analysis of unitary pseudogenes: historic and contemporary gene losses in humans and other primates
Source: Genome Biol. 2010 Mar 8;11(3):R26. doi: 10.1186/gb-2010-11-3-r26 (PMC2864566; doi:10.1186/gb-2010-11-3-r26)
Supplement: Additional file 1 — This file contains seven supplementary tables showing detailed results and datasets used in this study. [file gb-2010-11-3-r26-S1.PDF]

SUPPLEMENTARY MATERIAL

FOR

Identification and analysis of unitary pseudogenes: historic and contemporary gene losses in humans and other primates

Zhengdong D. Zhang [1], Adam Frankish [2], Toby Hunt [2], Jennifer Harrow [2], Mark Gerstein [1, 3, 4, §]

[1] Department of Molecular Biophysics and Biochemistry,
Yale University, New Haven, CT 06520, USA
[2] Wellcome Trust Sanger Institute,
Hinxton, Cambridgeshire CB10 1HH, England
[3] Interdepartmental Program in Computational Biology and Bioinformatics,
[4] Department of Computer Science,
Yale University, New Haven, CT 06520, USA

[§] Corresponding author (E-mail: mark.gerstein@yale.edu)

+

Supplementary tables

Table S1. Human pseudogenes of Olfactory receptors, vomeronasal receptors, zinc finger proteins, and their mouse orthologs.

Human unitary pseudogene genomic location	Mouse gene		
	MGI ID	Symbol	Name
chr1+:111198085-111199020	MGI:3030100	Olfr266	Olfactory receptor 266
chr1+:145357399-145358219	MGI:3031236	Olfr1402	Olfactory receptor 1402
chr1+:157587508-157588438	MGI:3031240	Olfr1406	Olfactory receptor 1406
chr1+:246004836-246005753	MGI:3030300	Olfr466	Olfactory receptor 466
chr2+:240697193-240698125	MGI:3031245	Olfr1411	Olfactory receptor 1411
chr3+:99266007-99266925	MGI:3030033	Olfr199	Olfactory receptor 199
chr3+:99513454-99514375	MGI:3030021	Olfr187	Olfactory receptor 187
chr5−:177196027-177197272	MGI:1333750	Olfr54	Olfactory receptor 54
chr6+:28052908-28053827	MGI:3031196	Olfr1362	Olfactory receptor 1362
chr6+:28109690-28110598	MGI:3031198	Olfr1364	Olfactory receptor 1364
chr6+:28122192-28123110	MGI:3031199	Olfr1365	Olfactory receptor 1365
chr6+:29366480-29367389	MGI:3030057	Olfr223	Olfactory receptor 223
chr6+:29493025-29493935	MGI:2177488	Olfr105	Olfactory receptor 105
chr6−:28129004-28531913	MGI:3031201	Olfr1367	Olfactory receptor 1367
chr6−:29102449-29103364	MGI:3031202	Olfr1368	Olfactory receptor 1368
chr6−:29213640-29214573	MGI:2177519	Olfr136	Olfactory receptor 136
chr6−:29304990-29305908	MGI:2177506	Olfr123	Olfactory receptor 123
chr7+:141233418-141234359	MGI:3030295	Olfr461	Olfactory receptor 461
chr7+:143308932-143309859	MGI:3030284	Olfr450	Olfactory receptor 450
chr7+:143470036-143470965	MGI:3030271	Olfr437	Olfactory receptor 437
chr7−:142895681-142896612	MGI:3030291	Olfr457	Olfactory receptor 457
chr9−:35849044-35849978	MGI:1860079	Olfr70	Olfactory receptor 70
chr10+:45073640-45074575	MGI:3030047	Olfr213	Olfactory receptor 213
chr11+:123430711-123431644	MGI:3030810	Olfr976	Olfactory receptor 976
chr11+:123534339-123535271	MGI:2660716	Olfr149	Olfactory receptor 149
chr11+:123583101-123584018	MGI:3030801	Olfr967	Olfactory receptor 967
chr11+:123600648-123601570	MGI:3030804	Olfr970	Olfactory receptor 970
chr11+:4356089-4357031	MGI:3030383	Olfr549	Olfactory receptor 549
chr11+:4395876-4396809	MGI:3030384	Olfr550	Olfactory receptor 550
chr11+:5070483-5071415	MGI:3030428	Olfr594	Olfactory receptor 594
chr11+:55033782-55034706	MGI:3031065	Olfr1231	Olfactory receptor 1231
chr11+:55379621-55380571	MGI:3030994	Olfr1160	Olfactory receptor 1160
chr11+:55595164-55596090	MGI:3030940	Olfr1106	Olfactory receptor 1106
chr11+:55606854-55607789	MGI:3030939	Olfr1105	Olfactory receptor 1105
chr11+:55936797-55937733	MGI:3030876	Olfr1042	Olfactory receptor 1042
chr11+:56152979-56153955	MGI:3030858	Olfr1024	Olfactory receptor 1024
chr11+:56561586-56562504	MGI:3030822	Olfr988	Olfactory receptor 988
chr11+:5697088-5698016	MGI:3030488	Olfr654	Olfactory receptor 654
chr11+:5704285-5705268	MGI:3030490	Olfr656	Olfactory receptor 656
chr11+:57668529-57669464	MGI:3031338	Olfr1504	Olfactory receptor 1504

chr11+:5778171-5779110	MGI:3030499	Olfr665	Olfactory receptor 665
chr11+:5851816-5852693	MGI:3030510	Olfr676	Olfactory receptor 676
chr11+:58981011-59273624	MGI:3031252	Olfr1418	Olfactory receptor 1418
chr11+:7724114-7725030	MGI:3030315	Olfr481	Olfactory receptor 481
chr11+:7826824-7827750	MGI:3030347	Olfr513	Olfactory receptor 513
chr11−:123480997-123481886	MGI:2660713	Olfr148	Olfactory receptor 148
chr11−:123740729-123741658	MGI:3030754	Olfr920	Olfactory receptor 920
chr11−:123751987-123752944	MGI:3030733	Olfr899	Olfactory receptor 899
chr11−:123856116-123857035	MGI:3030711	Olfr877	Olfactory receptor 877
chr11−:4124108-4125102	MGI:3030377	Olfr543	Olfactory receptor 543
chr11−:4492773-4493749	MGI:3030387	Olfr553	Olfactory receptor 553
chr11−:4638648-4639594	MGI:3030393	Olfr559	Olfactory receptor 559
chr11−:48410349-48411276	MGI:3031091	Olfr1257	Olfactory receptor 1257
chr11−:4950753-4951700	MGI:3030420	Olfr586	Olfactory receptor 586
chr11−:5053937-5054891	MGI:3030427	Olfr593	Olfactory receptor 593
chr11−:51292084-51292980	MGI:3031076	Olfr1242	Olfactory receptor 1242
chr11−:51383481-51384362	MGI:1333765	Olfr48	Olfactory receptor 48
chr11−:5148173-5149085	MGI:3030467	Olfr633	Olfactory receptor 633
chr11−:5408472-5409409	MGI:3030473	Olfr639	Olfactory receptor 639
chr11−:5446296-5447229	MGI:3030477	Olfr643	Olfactory receptor 643
chr11−:5529481-5530403	MGI:3030483	Olfr649	Olfactory receptor 649
chr11−:55427403-55428331	MGI:3030962	Olfr1128	Olfactory receptor 1128
chr11−:55712396-55713340	MGI:3030889	Olfr1055	Olfactory receptor 1055
chr11−:56121494-56122415	MGI:3030860	Olfr1026	Olfactory receptor 1026
chr11−:56193076-56193997	MGI:3030852	Olfr1018	Olfactory receptor 1018
chr11−:56325930-56326847	MGI:3030836	Olfr1002	Olfactory receptor 1002
chr11−:56343629-56344569	MGI:3030832	Olfr998	Olfactory receptor 998
chr11−:57889895-57890822	MGI:3031295	Olfr1461	Olfactory receptor 1461
chr11−:6044701-6045599	MGI:3030520	Olfr686	Olfactory receptor 686
chr11−:74459824-74460785	MGI:3030355	Olfr521	Olfactory receptor 521
chr11−:7751017-7751938	MGI:3030307	Olfr473	Olfactory receptor 473
chr12+:47307933-47308848	MGI:3030068	Olfr234	Olfactory receptor 234
chr12+:53795606-53796520	MGI:3030661	Olfr827	Olfactory receptor 827
chr12+:53838899-53902003	MGI:3030657	Olfr823	Olfactory receptor 823
chr12+:53942591-53943489	MGI:3030656	Olfr822	Olfactory receptor 822
chr12+:53963802-53964723	MGI:3030652	Olfr818	Olfactory receptor 818
chr12+:53991887-53992809	MGI:3030621	Olfr787	Olfactory receptor 787
chr12+:54068497-54069429	MGI:3030626	Olfr792	Olfactory receptor 792
chr12+:54202645-54255393	MGI:3030602	Olfr768	Olfactory receptor 768
chr12−:47122090-47122998	MGI:3030119	Olfr285	Olfactory receptor 285
chr12−:53873919-53874844	MGI:3030081	Olfr247	Olfactory receptor 247
chr12−:54056874-54057788	MGI:3030641	Olfr807	Olfactory receptor 807
chr13−:40903413-40904347	MGI:3030700	Olfr866	Olfactory receptor 866
chr14+:19385367-19386305	MGI:3030565	Olfr731	Olfactory receptor 731
chr14+:22241193-22242112	MGI:1333764	Olfr49	Olfactory receptor 49
chr14−:19406227-19407131	MGI:3031133	Olfr1299	Olfactory receptor 1299
chr14−:21208048-21208991	MGI:3031341	Olfr1507	Olfactory receptor 1507
chr15+:100186468-100187403	MGI:3031147	Olfr1313	Olfactory receptor 1313
chr15+:100206372-100207305	MGI:3031139	Olfr1305	Olfactory receptor 1305

chr15−:100284526-100285434	MGI:3031113	Olfr1279	Olfactory receptor 1279
chr17+:3160356-3161973	MGI:2177522	Olfr139	Olfactory receptor 139
chr19+:14888229-14899843	MGI:3031185	Olfr1351	Olfactory receptor 1351
chr19+:9175994-9176909	MGI:3030694	Olfr860	Olfactory receptor 860
chr19−:9250119-9251034	MGI:3030704	Olfr870	Olfactory receptor 870
chrX+:130165754-130166663	MGI:3031154	Olfr1320	Olfactory receptor 1320
chrX+:130385930-130386876	MGI:3031158	Olfr1324	Olfactory receptor 1324
chrX−:130286050-130286939	MGI:3031156	Olfr1322	Olfactory receptor 1322
chrX−:130359729-130360628	MGI:3031157	Olfr1323	Olfactory receptor 1323
chr3+:13943348-13944241	MGI:2148511	V1ra6	vomeronasal 1 receptor, A6
chr3−:14105594-14106381	MGI:2148510	V1ra5	vomeronasal 1 receptor, A5
chr6+:27159027-27159945	MGI:2159664	V1rh6	vomeronasal 1 receptor, H6
chr6−:27109204-27110093	MGI:2159690	V1ri4	vomeronasal 1 receptor, I4
chr7+:62443654-62476057	MGI:2148523	V1rc2	vomeronasal 1 receptor, C2
chr7+:62475153-62475952	MGI:2148530	V1rc9	vomeronasal 1 receptor, C9
chr7−:57486597-57486798	MGI:2159655	V1rg11	vomeronasal 1 receptor, G11
chr7−:62499330-62500076	MGI:2148508	V1ra3	vomeronasal 1 receptor, A3
chr7−:63032345-63033145	MGI:2159455	V1rc19	vomeronasal 1 receptor, C19
chr7−:63247448-63248334	MGI:3644380	V1rc31	vomeronasal 1 receptor, C31
chr7−:63541592-63542490	MGI:2148526	V1rc5	vomeronasal 1 receptor, C5
chr9+:39016648-39029593	MGI:3647050	V2r60	vomeronasal 2, receptor 60
chr9+:47053507-47054622	MGI:1316662	V2r88	vomeronasal 2, receptor 88
chr9−:40361841-40374789	MGI:3644540	V2r99	vomeronasal 2, receptor 99
chr11−:57565222-57565526	MGI:1351346	V2r57	vomeronasal 2, receptor, 57
chr16+:31465703-31466530	MGI:2159654	V1rg10	vomeronasal 1 receptor, G10
chr16+:31567162-31568045	MGI:3645555	V1rc29	vomeronasal 1 receptor, C29
chr16−:31726776-31727667	MGI:2159642	V1rg3	vomeronasal 1 receptor, G3
chr19+:58510203-58511091	MGI:2159699	V1rl1	vomeronasal 1 receptor, L1
chr19−:45319222-45319978	MGI:2159646	V1rg7	vomeronasal 1 receptor, G7
chr19−:61520313-61521188	MGI:3642986	V2r8	vomeronasal 2, receptor 8
chr19−:63215743-63229149	MGI:3644480	V2r53	vomeronasal 2, receptor 53
chrX+:48379793-48380315	MGI:2148515	V1rb1	vomeronasal 1 receptor, B1
chr18+:31125147-31141583	MGI:99179	Zfp35	zinc finger protein 35

Table S2. Human unitary pseudogenes and their mouse orthologs.

Human unitary pseudogene genomic location	Mouse gene MGI ID	Mouse gene symbol	Mouse gene name	Reference
chr12+:110821507-110823878	MGI:2429506	Adam1b	a disintegrin and metallopeptidase domain 1b	[48]
chr8+:17371392-17373372	MGI:3588304	Adam26B	a disintegrin and metallopeptidase domain 26B	
chr8−:39450156-39489335	MGI:102518	Adam3	a disintegrin and metallopeptidase domain 3 (cyritestin)	[48]
chr8+:39299218-39358412	MGI:104730	Adam5	a disintegrin and metallopeptidase domain 5	[48]
chr9−:103136199-103141451	MGI:2444345	Acnat2	acyl-coenzyme A amino acid N-acyltransferase 2	[49]
chr18+:54814947-54887164	MGI:2442915	Acyl3	acyltransferase 3 [RIKEN cDNA 5330437I02 gene]	[23]
chr1+:92304452-92305907	MGI:1918152	Aytl1b	acyltransferase like 1B	
chr11+:71909632-71910345	MGI:107545	Art2b	ADP-ribosyltransferase 2b	[50]
chr2+:201166115-201364602	MGI:3529596	Aox3l1	aldehyde oxidase 3-like 1	[51]
chr16+:2351147-2415839	MGI:3625331	Abca17	ATP-binding cassette, sub-family A (ABC1), member 17	[52]
chr1−:51789487-51812353	MGI:2140435	Calr4	calreticulin 4	
chr16−:30823174-30826438	MGI:2684607	Ctf2	cardiotrophin 2	[20]
chr4−:123871155-123872802	MGI:2677454	Cetn4	centrin 4	
chr19−:46006279-46009136	MGI:2686296	Cyp2t4	cytochrome P450, family 2, subfamily t, polypeptide 4	
chr2−:178665477-178677441	MGI:88579	Cyct	cytochrome c, testis	[2]
chr4−:68540001-68564082	MGI:2444058	Desc4	Desc4 [RIKEN cDNA 9930032O22 gene]	
chr11−:67136888-67140266	MGI:1926250	Doc2g	double C2, gamma	
chr9+:35423704-35439561	MGI:2444287	Feta	Feta [RIKEN cDNA 4930417M19 gene]	
chr10−:114057930-114106344	MGI:106025	Gucy2g	guanylate cyclase 2g	
chr8:27473706-27502505	MGI:1353434	Gulo	gulonolactone (L-) oxidase	[19]
chr1−:226718541-226718916	MGI:1925553	Hist3h2ba	histone cluster 3, H2ba	
chr7+:123241442-123256569	MGI:1921659	Hyal6	hyaluronoglucosaminidase 6	[53]
chr9−:114761447-114764366	MGI:97236	Mup4	major urinary protein 4	[24]
chr10+:81670064-81672769	MGI:96923	Mbl1	mannose binding lectin (A) 1	[54]
chr6+:118061593-118072916	MGI:1913900	Nepn	nephrocan	
chr3+:47028800-47029644	MGI:1914419	Nradd	neurotrophin receptor associated death domain	
chr1+:115181467-115195621	MGI:3026618	Nr1h5	nuclear receptor subfamily 1, group H, member 5	[4]
chrX+:101400687-101403403	MGI:1923079	Prame	preferentially expressed antigen in melanoma	[55]
chr1+:200404371-200425048	MGI:108027	Ptprv	protein tyrosine phosphatase, receptor type, V	
chr5+:140786050-140870922	MGI:1935200	Pcdhgb8	protocadherin gamma subfamily B, 8	
chr19+:53875091-53876096	MGI:1928893	Sec1	secretory blood group 1	[56]
chr20−:1696610-1708642	MGI:3045317	Sirpb3	Sirpb3 [RIKEN cDNA F830045P16 gene]	
chr2+:20449670-20459798	MGI:3045351	Slc7a15	solute carrier family 7 (cationic amino acid transporter, y+ system), member 15	
chr4−:70692183-70714196	MGI:1926341	Sult1d1	sulfotransferase family 1D, member 1	[57]
chr7+:142844251-142845153	MGI:2681300	Tas2r134	taste receptor, type 2, member 134	
chr17+:59285910-59292052	MGI:1923120	Tcam1	testicular cell adhesion molecule 1	
chrX+:83901067-83903982	MGI:1890545	Tex16	testis expressed gene 16	
chr14−:63882652-63893934	MGI:1931131	Tex21	testis expressed gene 21	
chr8−:145268106-145414584	MGI:1920792	Tssk5	testis-specific serine kinase 5	[58]
chr17−:73756179-73757460	MGI:1919026	Tha1	threonine aldolase 1	[59]
chr1+:33704438-33707143	MGI:3045221	Tlr12	toll-like receptor 12	[60]

chr6:−132971083-132972109	MGI:3527427	Taar3	trace amine-associated receptor 3	[61]
chr6−:132957230-132958269	MGI:2685072	Taar4	trace amine-associated receptor 4	[61]
chr11+:3587708-3615320	MGI:109527	Trpc2	transient receptor potential cation channel, subfamily C, member 2	[62]
chr4−:68314827-68322204	MGI:3521861	Tmprss11c	transmembrane protease, serine 11c	
chr16−:2829662-2831734	MGI:1353645	Tmprss8	transmembrane protease, serine 8 (intestinal)	
chr1−:84603696-84623086	MGI:98907	Uox	urate oxidase	[15]
chr20+:31371468-31377322	MGI:2385160		cDNA sequence BC018465 [MGC27784]	
chr1−:176243843-176270560	MGI:2448516		cDNA sequence BC026585	
chr5−:54139705-54164798	MGI:3040697		cDNA sequence BC067074	
chr3−:15186506-15194712	MGI:3648943		RIKEN cDNA 1110001D15 gene	
chr12+:54512211-54513592	MGI:1921077		RIKEN cDNA 1110012D08 gene	
chr22+:40678116-40683782	MGI:1923755		RIKEN cDNA 1500009C09 gene	
chr8−:143647874-143652195	MGI:1916689		RIKEN cDNA 1700016M24 gene [MGC132932]	
chr17−:40680535-40682512	MGI:1916691		RIKEN cDNA 1700023F06 gene [MGC118352]	
chr2+:233096815-233098443	MGI:1916703		RIKEN cDNA 1700027L20 gene	
chr7−:141612140-141617641	MGI:1920731		RIKEN cDNA 1700074P13 gene	
chr4−:1099052-1137515	MGI:1914616		RIKEN cDNA 1810008K16 gene	
chr1+:199168021-199196188	MGI:1923672		RIKEN cDNA 2310006M14 gene	
chr5−:102794203-102892036	MGI:1916489		RIKEN cDNA 2610034M16 gene	
chr11−:67176299-67177402	MGI:1919908		RIKEN cDNA 2700097O09 gene	
chr3−:49885785-49889743	MGI:1914972		RIKEN cDNA 4921517D21 gene [MGC141498]	
chr13+:30549392-30587839	MGI:1923042		RIKEN cDNA 4930434E21 gene	
chr1+:108617285-108681521	MGI:1921936		RIKEN cDNA 4930443G12 gene	
chr7−:91880233-91893753	MGI:1922305		RIKEN cDNA 4930511M11 gene	
chrX+:84115399-84119300	MGI:1918280		RIKEN cDNA 4933403O08 gene	
chr17+:70581997-70585413	MGI:3588186		RIKEN cDNA 4933422H20 gene	
chr5−:63736096-63782410	MGI:1914013		RIKEN cDNA 4933425L06 gene [MGC141320]	
chr16+:22301259-22326251	MGI:1921716		RIKEN cDNA 4933427G17 gene	
chrX−:77307850-77314627	MGI:2685621		RIKEN cDNA 5031408O05 gene	
chr3+:169450424-170021809	MGI:1922990		RIKEN cDNA 6130401L20 gene	
chr19+:1475139-1485047	MGI:3026984		RIKEN cDNA 6330514A18 gene [MGC106082]	
chr20+:23404347-23408436	MGI:1925859		RIKEN cDNA 8030411F24 gene	
chr18+:8449158-8503840	MGI:1921806		RIKEN cDNA 9130404H23 gene	
chr5−:139929821-139951871	MGI:3687212		RIKEN cDNA E230025N22 gene	
chr11+:43534868-43548045	MGI:3041234		RIKEN cDNA E530001K10 gene	

Table S3. Time estimates of several pseudogenization events that occurred in the human lineage after the human-chimp divergence.

Gene symbol	Pseudogenization time (MYA[1])	Standard error (MYA)
MUP	0.27	4.42
TMPRSS8	0.32	1.32
CTF2	1.93	4.49
ADAM5	2.10	1.23
HYAL6	2.53	5.44
1700074P13Rik	2.55	5.02
MBL1	4.24	3.26
4930443G12Rik	6.10	2.22

1. MYA: million years ago

Table S4. Polymorphic pseudogenes with the disruptive sites typed in the HapMap Project. [1]

CDS-disrupted gene	GPR33	SERPINB11	TAAR9
Disruptive mutation [2]	Cga (R) → Tga	Gaa (E) → Taa	Aaa (K) → Taa
dbSNP ID	rs17097921	rs4940595	rs2842899
Genomic location	chr14−:31,022,505	chr18+:59,530,818	chr6+:132,901,302
Disrupted codon position [3]	140 (332)	89 (388)	61 (344)
Reference allele in human	T	T	T
Reference allele in other primates [4]	C	T	T
Allele frequency [5]	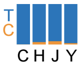	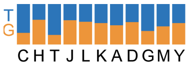	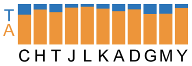
Test statistic for HWE in the meta-population [6]	0.285 (P = 0.867)	8.659 (P = 0.013)	0.071 (P = 0.965)

1. This is the full version of Table 3, showing the allele frequency information.
2. Both codons before and after the mutation (→) are shown with the affected base capitalized. The amino acid residue encoded by the codon is given in the parentheses.
3. The disrupted codon position in the coding sequence (CDS). The number of codons in the CDS is given in the parentheses.
4. Widely regarded as the ancestral allele. Other primates currently include chimp, orangutan, and macaque.
5. Allele frequency data are currently available for up to 11 populations from the HapMap Project. The one-letter population code, which is used for brevity, has the following meaning:

A	ASW	African ancestry in Southwest USA
C	CEU	Utah residents with Northern and Western European ancestry from the CEPH collection
H	CHB	Han Chinese in Beijing, China
D	CHD	Chinese in Metropolitan Denver, Colorado
G	GIH	Gujarati Indians in Houston, Texas
J	JPT	Japanese in Tokyo, Japan
L	LWK	Luhya in Webuye, Kenya
M	MEX	Mexican ancestry in Los Angeles, California
K	MKK	Maasai in Kinyawa, Kenya
T	TSI	Toscans in Italy
Y	YRI	Yoruba in Ibadan, Nigeria

6. The χ^2 goodness-of-fit test is used to test for the Hardy-Weinberg equilibrium in the meta-population using the pooled genotype and allele frequency data.

Table S5. Comparison with gene losses reported by Zhu et al. (2007). [1]

HG18 Location	Mouse annotation (if any)			
	Gene symbol	MGI ID	Gene name	
I. Present in our set				
chr18:54814918-54887549	*Acyl3*	MGI:1918152	Acyltransferase 3	
chr20:31368330-31384061	*BC018465*	MGI:2385160	Hypothetical protein LOC228802	
chr4:123870718-123873126	*Cetn4*	MGI:2677454	Centrin 4	
chr11:243417-243558	*Cox8b*	MGI:105958	Cytochrome 8, subunit VIIb	
chr16:30822866-30829066	*Ctf2*	MGI:2684607	Cardiotrophin 2	
chr12:22135947-22179281	*Gm766*	MGI:2685612	Hypothetical protein LOC330440	
chr8:27473714-27503386	*Gulo*	MGI:1353434	Gulonolactone (L-) oxidase	
chr6:118061568-118080190	*Nepn*	MGI:1913900	Hypothetical protein LOC66650	
chr3:47026634-47029966	*Nradd*	MGI:1914419	Death domain containing membrane protein NRADD	
chr2:20455521-20460982	*Slc7a15*	MGI:3045351	Aromatic-preferring amino acid transporter	
chr4:70687437-70720060	*Sult1d1*	MGI:1926341	Sulfotransferase family 1D, member 1	
chr6:132957223-132958268	*Taar4*	MGI:2685072	Trace amine-associated receptor 4	
chr14:63879902-63915769	*Tex21*	MGI:1931131	Testis expressed gene 21	
chr1:84603188-84636156	*Uox*	MGI:98907	Urate oxidase	
chr11:32089736-32311979	*0610012H03Rik*	MGI:1921338	Hypothetical protein LOC74088	
chr3:185699692-185701043	*2310042E22Rik*	MGI:1913811	Hypothetical protein LOC66561	
chr14:34493816-34521518	*2700097O09Rik*	MGI:1919908	Hypothetical protein LOC72658	
chr3:49885419-49889756	*4921517D21Rik*	MGI:1914972	Hypothetical protein LOC67722	
II. Absent from our set				
II.1 Human ortholog by Inparanoid exists				*Human ortholog Ensembl protein ID*
chr2:209718174-209720719	*Crygf*	MGI:88526	Crystallin, gamma F	ENSP00000264376
chr19:46247785-46260189	*Cyp2g1*	MGI:109612	Cytochrome P450, family 2, subfamily g	ENSP00000373426
chr17:20356269-20361411	*Krt1-17*	MGI:96691	Keratin complex 1, acidic, gene 17	ENSP00000319235
chr20:31397095-31406542	*LOC433492*	MGI:1918675	Hypothetical protein 5430413K10Rik	ENSP00000246222
chr12:55661222-55666095	*Rdh7*	MGI:1860517	Retinol dehydrogenase 7	ENSP00000266988
chr4:70142918-70151231	*Ugt2a3*	MGI:1919344	UDP glucuronosyltransferase 2 family a3	ENSP00000251566
chr4:69904790-69920682	*Ugt2b1*	MGI:1919023	UDP glucuronosyltransferase 2 family b1	ENSP00000305221
chr17:19423766-19477673	*4933429E10Rik*	MGI:3588190	Hypothetical protein LOC380701	ENSP00000270570
II.2 Mouse protein sequence cannot be aligned to the human genome				
chr1:22149751-22152304	*1700013G24Rik*	MGI:1916630	Hypothetical protein LOC69380	
chr6:41257342-41262067	*B430306N03Rik*	MGI:2443478	Hypothetical protein LOC320148	
chr12:53163432-53172287	*BC048502*	MGI:2652828	Hypothetical protein LOC223927	
chr9:122,649,393-122,655,217	*D730039F16Rik*	MGI:1925246	Contain CutA1 domain	
II.3 No disablement is found in the best alignment of the mouse gene to the human genome				
chr16:21857687-21863975	*Abca14*	MGI:2388708	ATP-binding cassette, sub-family A, member 14	

chr19:15535443-15556847	*Cyp4f16*	MGI:1917351	Cytochrome P450, family 4, subfamily f, polypeptide 16
chr6:52912485-52945892	*Gsta4*	MGI:1309515	Glutathione S-transferase, alpha 4
chr21:30730324-30731174	*Krtap14*	MGI:1346079	Keratin associated protein 14
chr17:36622484-36623047	*Krtap9-1*	MGI:1309997	Keratin associated protein 9-1
chr11:58810633-58814011	*Pfpl*	MGI:1860266	Pore forming protein-like
chr16:2789020-2795066	*Prss32*	MGI:1917064	Tryptase 5
chr22:20336505-20345277	*2610318N02Rik*	MGI:1917708	Hypothetical protein LOC70458
chr12:51088354-51101918	*4732456N10Rik*	MGI:3045312	Similar to keratin, type II cytoskeletal 6D
II.4 Mouse protein sequence is not included in the Inparanoid mouse set			
chr11:76074935-76084205	*Gucy2d*	MGI:106030	Hypothetical protein LOC434214
II.5 Duplicated pseudogene			
chr4:74932359-74933982	*Cxcl7*	MGI:1888712	Pro-platelet basic protein
chr14:31021882-31026992	*Gpr33*	MGI:1277106	G protein-coupled receptor 33
chr15:42905239-42941396	*Sord*	MGI:98266	Sorbitol dehydrogenase 1
chr1:151635327-151638220	*S100a15*	MGI:1913416	S100 calcium binding protein A15
chr6:167541894-167555675	*Unc93a*	MGI:1933250	Unc-93 homolog A

1. Based on and modified from Table S4 from [23]. Olfactory receptor pseudogenes are omitted.

Table S6. Previously identified human unitary pseudogenes absent from this study.

Gene symbol	Gene Name	References
ABCA14	ATP-binding cassette, sub-family A, member 14	[63]
CMAH	CMP-N-acetylneuraminic acid hydroxylase	[12]
CRYGF	Crystallin, gamma F	[64]
CYP2G1	Cytochrome P450, family 2, subfamily g	[65]
GSTA4	Glutathione S-transferase, alpha 4	[66]
MYH16	Myosin heavy chain 16	[14]

Table S7. Genome sequences used for ortholog identification.

Organism	Genome assembly	Release date
Human	hg18	Mar. 2006
Chimp	panTro2	Mar. 2006
Gorilla	gorGor1	Oct. 2008
Orangutan	ponAbe2	Jul. 2007
Rhesus	rheMac2	Jan. 2006
Marmoset	calJac1	Jun. 2007
Tarsier	tarSyr1	Aug. 2008
Mouse lemur	micMur1	Jun. 2003
Bushbaby	otoGar1	Dec. 2006
TreeShrew	tupBel1	Dec. 2006
Mouse	mm9	Jul. 2007
Rat	rn4	Nov. 2004
Kangaroo rat	dipOrd1	Jul. 2008
Guinea Pig	cavPor3	Feb. 2008
Squirrel	speTri1	Feb. 2008
Rabbit	oryCun1	May 2005
Pika	ochPri2	Jul. 2008
Alpaca	vicPac1	Jul. 2008
Dolphin	turTru1	Feb. 2008
Cow	bosTau4	Oct. 2007
Horse	equCab2	Sep. 2007
Cat	felCat3	Mar. 2006
Dog	canFam2	May 2005
Microbat	myoLuc1	Mar. 2006
Megabat	pteVam1	Jul. 2008
Hedgehog	eriEur1	Jun. 2006
Shrew	sorAra1	Jun. 2006
Elephant	loxAfr2	Jul. 2008
Tenrec	echTel1	Jul. 2005
Armadillo	dasNov2	Jul. 2008
Opossum	monDom4	Jan. 2006
Platypus	ornAna1	Mar. 2007
Chicken	galGal3	May 2006
Zebra finch	taeGut1	Jul. 2008
Lizard	anoCar1	Feb. 2007
X. tropicalis	xenTro2	Aug. 2005
Tetraodon	tetNig1	Feb. 2004
Fugu	fr2	Oct. 2004
Stickleback	gasAcu1	Feb. 2006
Medaka	oryLat2	Oct. 2005
Zebrafish	danRer5	Jul. 2007
Lamprey	petMar1	Mar. 2007
